# Enhancing Mentoring in Palliative Care: An Evidence Based Mentoring Framework

**DOI:** 10.1177/2382120520957649

**Published:** 2020-09-23

**Authors:** Lalit Kumar Radha Krishna, Lorraine Hui En Tan, Yun Ting Ong, Kuang Teck Tay, Jia Min Hee, Min Chiam, Elisha Wan Ying Chia, Krish Sheri, Xiu Hui Tan, Yao Hao Teo, Cheryl Shumin Kow, Stephen Mason, Ying Pin Toh

**Affiliations:** 1 Yong Loo Lin School of Medicine, National University of Singapore, Singapore; 2 Division of Supportive and Palliative Care, National Cancer Centre Singapore, Singapore; 3 The Palliative Care Centre for Excellence in Research and Education, Singapore; 4 Division of Cancer Education, National Cancer Centre Singapore, Singapore; 5 Palliative Care Institute Liverpool, Academic Palliative & End of Life Care Centre, University of Liverpool, Liverpool, UK; 6 Duke-NUS Medical School, National University of Singapore, Singapore; 7 Centre of Biomedical Ethics, National University of Singapore, Singapore; 8 National University Hospital, National University Health System, Singapore

**Keywords:** Palliative care, palliative care education, mentoring, medicine, novice mentoring, medical school, postgraduate medicine

## Abstract

**Background:**

Growing concerns over ethical issues in mentoring in medicine and surgery have hindered efforts to reinitiate mentoring for Palliative Care (PC) physicians following the easing of COVID-19 restrictions. Ranging from the misappropriation of mentee’s work to bullying, ethical issues in mentoring are attributed to poor understanding and structuring of mentoring programs, underlining the need for a consistent approach to mentoring practices.

**Methods:**

Given diverse practices across different settings and the employ of various methodologies, a novel approach to narrative reviews (NR)s is proposed to summarize, interpret, and critique prevailing data on novice mentoring. To overcome prevailing concerns surrounding the reproducibility and transparency of narrative reviews, the Systematic Evidenced Based Approach (SEBA) adopts a structured approach to searching and summarizing the included articles and employed concurrent content and thematic analysis that was overseen by a team of experts.

**Results:**

A total of 18 915 abstracts were reviewed, 62 full text articles evaluated and 41 articles included. Ten themes/categories were ascertained identified including Nature; Stakeholders; Relationship; Approach; Environment; Benefits; Barriers; Assessments; Theories and Definitions.

**Conclusion:**

By compiling and scrutinizing prevailing practice it is possible to appreciate the notion of the mentoring ecosystem which sees each mentee, mentor, and host organization brings with them their own microenvironment that contains their respective goals, abilities, and contextual considerations. Built around competency based mentoring stages, it is possible to advance a flexible yet consistent novice mentoring framework.

## Background

Novice Mentoring in Palliative Care (PC), enhances mentees’ clinical skills, inculcates appropriate attitudes, and practices in caring for dying patients and advances the reputation of the host organization.^1-3^ Characterized as a “*dynamic*, *context dependent*, *goal sensitive*, *mutually beneficial relationship between an experienced clinician (mentor) and junior clinicians and/or under-graduates (mentee) that is focused upon advancing the development of the mentee*,”^4^ novice mentoring is increasingly used in the training of PC residents and specialist trainees.

However inertia to the resumption of novice mentoring programs following the loosening of COVID-19 restrictions has caught many off guard. At the heart of concerns amongst administrators, program designers, and curriculum advisor as well as some mentors and mentees are growing concerns over reports of ethical issues in mentoring.^5-14^ Attributed to poor understanding and consequently ineffective structuring of mentoring programs recent reports list misappropriation of mentees’ work, breaching professional boundaries, and bullying as just some of the issues faced by mentees in poorly structured and supported programs.^5-14^ Addressing gaps in understanding and structuring novice mentoring is hampered by a lack of data.

### Problems in researching novice mentoring

To address this lacuna and better inform the structuring of mentoring programs, review of novice mentoring data is required. However such an endeavor must acknowledge the fact that prevailing data involves a variety of methodologies and practices and does not often consider mentoring’s evolving nature that requires a longitudinal perspective of mentoring nor its context specific nature that limits comparisons of mentoring data across different research, clinical, academic, practice and healthcare settings, and mentoring goals. Scrutiny of novice mentoring is also limited by poorly defined terms and the mistaken intermixing of mentoring and educational approaches such as peer, near-peer, group, leadership, patient, family, and e-mentoring as well as advising, sponsoring, role modeling, tutoring, coaching, supervising, and networking which have their own distinct approaches and roles in medical education. Such conflation of terms and practices also compromises structured search processes which relies on clearly defined terms to guide the search process. In addition many review fail to consider the socially constructed aspect of mentoring nor the need to include longitudinal mentoring experiences and perspectives of mentees, mentors, and other stakeholders that are often captured in gray literature. These considerations and the need for a multidimensional socioculturally informed perspective of mentoring from the viewpoint of mentees, mentors, the host organization, and other stakeholders also underline the need for a constructivist approach and the use of a relativist lens.

In the face of data suggesting that mentoring can be extrapolated from accounts of novice mentoring in Internal Medicine (IM),^15-18^ we will seek to study prevailing novice mentoring data in IM and extrapolate these findings to PC.

## Methods

With use of systematic and scoping reviews limited by poorly defined mentoring terms that will compromise structured searches, a narrative review (NR) is proposed given its ability to delve into the underlying^19^ and the hidden systems that drive the mentoring process^20-28^ across different research traditions.^21,26,27^ An NR is also better equipped to contend with data from gray literature and forward a better understanding of why^21^ and how mentoring’s socioculturally informed processes evolve and affect stakeholders and their mentoring relationships.^20,25-27,29-37^

To address concerns that NRs lack transparency, accountability, and structure in the synthesis of narratives, we adopt Krishna’s novel methodology called Systematic Evidenced Based Approach (SEBA). Built upon a constructivist perspective, a SEBA guided NR (henceforth NR in SEBA) is able to map complex topics from multiple angles^38^ whilst its relativist lens allows for the collation of historically, socioculturally, ideologically, and contextually influenced views, experiences and accounts of mentees, mentors, and host organizations (henceforth stakeholders).

Guided through each stage of the synthesis of an NR in SEBA by an expert team comprised of medical librarians from the Yong Loo Lin School of Medicine (YLLSoM) at the National University of Singapore and the National Cancer Centre Singapore (NCCS), and local educational experts and clinicians at the NCCS, the Palliative Care Institute Liverpool, YLLSoM, and Duke-NUS Medical School, and guided by the principles of interpretivist analysis, the research team immerse and chart their review, and analysis of the qualitative data. Team discussions and expert guidance ensured that the data was pieced together in a meaningful, transparent, and reproducible manner.^21,39-41^ This enhances the accountability of the search process.

**Figure 1.**
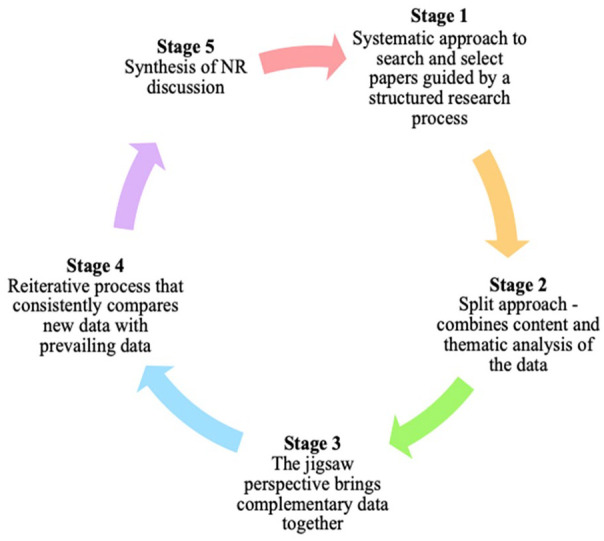
The SEBA process.

Outlined in Figure 1, the SEBA process comprises the following stages: (1) Systematic Approach, (2) Split Approach, (3) Jigsaw Perspective, (4) Reiterative Process, and (5) Synthesis of NR in SEBA. Each stage will be elaborated upon throughout the paper.

### Stage 1 of SEBA: Systematic approach

#### Determining the title and research question

Ensuring a systematic approach to the synthesis of NRs in SEBA, the expert and research team established the overall goals of the NR and the population, context, and concept to be evaluated. The research question was determined to be “*what is known of novice mentoring in Internal Medicine (henceforth IM)?*” In order to inform the design, structure, and oversight of novice mentoring programs, the secondary research questions were determined to be “*what are the key characteristics of novice mentoring in IM?*” and “*what processes contribute to their success?*”

#### Inclusion criteria

A Population Intervention Comparison Outcome Study Design (PICOS) format was adopted to guide the research process.^42,43^ This is outlined in Table 1.

**Table 1.** PICOS, inclusion and exclusion criteria.

PICOS	Inclusion criteria	Exclusion criteria
Population	• Junior physicians, residents, and medical students in IM specialties delineated by the American College of Physicians including Allergy and Immunology, Clinical Medicine, Community Medicine, Dermatology, General Practice, Geriatrics, Hospital Medicine, Neurology, Palliative Medicine, Cardiology, Endocrinology, Gastroenterology, Hematology, Immunology, Infectious Disease, Nephrology, Respiratory Medicine, and Rheumatology	• Clinical specialties not associated with medicine such as surgical specialties, Pediatrics, Emergency Medicine, Obstetrics and Gynecology, and Clinical and Translational Science
Intervention	• Systematic review or scoping reviews or systematic scoping reviews or narrative reviews of novice mentoring involving junior physicians, residents and/or medical students mentored by senior clinicians aimed at advancing the professional and/or personal development of the mentee ○ Mentoring processes ○ Mentor factors ○ Mentee factors ○ Mentoring relationship ○ Host organization ○ Outcomes of mentoring ○ Barriers to mentoring ○ Mentoring structure ○ Mentoring framework ○ Mentoring culture ○ Mentoring environment	• Peer mentoring, mentoring for leadership, mentoring patients or mentoring by patients, interdisciplinary mentoring • Supervision, coaching, role-modelling, advising, and sponsorship
Comparison	• Comparisons of accounts of mentoring between mentoring programs, editorials and perspective, reflective, narratives, and opinions pieces	
Outcome	• Personal outcomes of mentoring • Professional development outcomes • Career related outcomes • Research and academia outcomes	• Studies where mentoring outcomes were not the main component evaluated
Study design	• Systematic review, literature reviews, and narrative reviews • All study designs are included ○ Descriptive papers ○ Qualitative, quantitative, and mixed study methods • Perspectives, opinion, commentary pieces, and editorials • Published between 1st January 2000 to 31st December 2019	

#### Searching

Ten members of the research team carried out independent searches of 7 bibliographic databases (PubMed, Embase, PsycINFO, ERIC, Cochrane Database of Systematic Reviews, Google Scholar, and Scopus) and gray literature databases (GreyLit, OpenGrey, and Web of Science) between 17 December 2019 and 17 January 2020. Focus on mentoring in IM rather than PC was in acknowledgement of a dearth of mentoring data in PC and evidence that extrapolation of data from IM and PC is possible. The PubMed Search Terms and Strategy may be found in Supplemental Appendix 1.

#### Extracting and charting

Using an abstract screening tool, each member of the research team independently reviewed the titles and abstracts found in each database and drew up a list of titles to be reviewed. Comparing these individual lists via online meetings, Sambunjak et al’s^44^ approach to “negotiated consensual validation” was used to achieve consensus on the final list of titles to be reviewed. The research team then independently reviewed each full-text article from this final list, created individual lists for inclusion, held team discussions online and arrived at a consensus on the final list of full-text articles to be included in the NR in SEBA. To circumnavigate the limitations caused by poorly delineated mentoring terms, the research teams reviewed the references of the included articles and snowballing their findings to reduce the chance of omitting relevant articles.

### Stage 2 of SEBA: Split approach

Dividing into 3 teams, the researchers simultaneously reviewed the included full-text articles. The first team adopted Braun and Clarke’s^45^ approach to thematic analysis whilst the second team employed Hsieh and Shannon’s^46^ approach to directed content analysis. Used to enhance the trustworthiness of the results, concurrent thematic and content analysis is the hallmark of the Split Approach The third team summarized and tabulated the included articles, keeping with recommendations drawn from Wong et al’s^38^ RAMESES publication standards: meta-narrative reviews and Popay et al’s^47^ “Guidance on the conduct of narrative synthesis in systematic reviews.” The tabulated summaries serve to ensure that key aspects of the included articles are not lost. They are presented in Supplemental Appendix 2.

#### Braun and Clarke’s thematic analysis

A reiterative step-by-step thematic analysis was carried and this saw codes constructed from the “surface” meaning of the articles.^48-50^ These codes were then organized into themes deemed to best represent the whole data set.^51^ Each member independently reviewed and refined their themes with negotiated consensual validation used to establish a final list of themes.^52^

#### Hsieh and Shannon’s directed content analysis

Directed content analysis was employed to enhance validity of the themes identified and addressed the relative failure of Braun and Clarke’s approach in resolving contradictory data.^53^

Identifying and operationalizing a priori coding categories,^54^ reviewers drew codes from Krishna et al^’^s^17^ “*Mentoring stages: A study of undergraduate mentoring in palliative medicine in Singapore*,” the first clinically evidenced account of novice mentoring in Palliative Medicine. Deductive category application was employed to identify any relevant data not captured by existing codes and subsequently assigned a new code.^55^ Each member independently reviewed and refined their codes with negotiated consensual validation used to establish a final “code book.”

### Stage 3 of SEBA: Jigsaw perspective

The jigsaw perspective brings together complementary data and is especially important in ensuring that critical aspects are not lost in the “funneling” process that follows. This “funneling” process saw the themes and categories identified in the independent analyses compared and combined to present a holistic perspective of novice mentoring thus facilitating its effective analysis and interpretation.

### Stage 4 of SEBA: Reiterative process

As part of the reiterative process, the findings were discussed with the expert team and concerns were raised over the influence of gray literature on the results as they were neither peer reviewed nor clearly evidence based. This saw the research team differentiating gray literature such as correspondence, letters, editorials, and perspective pieces extracted from academic databases from data driven and research based peer reviewed articles. Both were thematically analyzed independently and the themes derived from the gray literature were found to be in agreement with themes from the peer-reviewed literature.

In total, there were 10 themes/categories revealed through Braun and Clarke’s and Hsieh and Shannon’s approach. As extensive overlaps were observed, they will be presented and discussed together.

## Results

A total of 18 915 abstracts were reviewed, 62 full-text articles evaluated and 41 articles included. This is outlined in the PRISMA Flowchart in Supplemental Appendix 3. The following themes/categories pertaining to novice mentoring was discerned: (1) Definitions, (2) Nature, (3) Stakeholders, (4) Relationship, (5) Approach, (6) Environment, (7) Benefits, (8) Barriers, (9) Assessments, and (10) Theories.

### Definitions

Wesley et al’s^56^ definition which dominates prevailing practice characterizes novice mentoring as “*an evolving relationship between an experienced clinician and junior clinicians and/or students that is focused upon creating personalised and enduring mutually beneficial mentoring relationships*.”

However 5 other considerations were highlighted:

(a) the host organization’s role in the mentoring relationship.
(b) the host organization’s role in nurturing the mentoring environment through the establishment of clear codes of conduct and provision of support for consistent, longitudinal and holistic assessments, matching processes, and training programs.
(c) the presence of mentoring stages in a structured mentoring process.
(d) the need to balance between a consistent mentoring approach, compliance with codes of practice, and flexibility so as to accommodate for evolving needs, goals, and expectations of stakeholders.
(e) novice mentoring’s evolving, goal-sensitive, context-specific, mentee-, mentor-, organizational- and relational-dependent nature and thus implication on research and support.

### Mentoring nature

As briefly outlined in the final consideration above, novice mentoring possesses critical characteristics further elaborated in Table 2.

**Table 2.** Elements of the nature of mentoring.

Elements of the nature of mentoring	Elaboration	References
Context-specific	Mentoring methods differ in clinical, research, and academic settings.	Ikbal et al^57^
	There are further differences in the undergraduate and postgraduate settings as a result of different goals, namely preparing students for medical school and piquing their interest in specialties, honing skills, and more holistic support in the 2 settings respectively.	Ikbal et al^57^; Qiao Ting Low et al^58^; Toh et al^59^
	These different goals and stakeholders may then lead to unique combinations of mentoring approaches, requirements, structures, and mentoring relationships.	Sng et al^15^; Ikbal et al^57^
Goal-sensitive	The mentoring relationship results in shifts in short-term objectives to achieve long-term goals.	Ikbal et al^57^
	These shifts illustrate how mentoring concerns itself with reaching goals set by mentees, mentors, and the host organization. It is also of note that long term goals may also evolve with time.	Toh et al^59^
Evolving	Mentoring is subject to changes in internal stakeholder dependent factors and external influences.	Ikbal et al^57^
	In addition to evolving goals, mentors and mentees need to “respond appropriately depending upon their situation, ability and motivations” as well as to “challenges and opportunities.”	Ikbal et al^57^
Stakeholder-dependent	Mentoring needs to meet mentees’ personal circumstances. This is further supported by evidence that mentoring differs in the undergraduate and the postgraduate setting.	Ikbal et al^57^
	The mentor’s ability to support the mentee and build an effective mentoring relationship influences the mentoring experience.	Ikbal et al^57^
	This is further evidenced by the different roles mentors play in different mentoring settings and different stages of mentoring.	Sng et al^15^; Ikbal et al^57^; Tohet al^59^
Approach-dependent	The mentoring process differs with variations in aspects of the process, be it in the initiation of the mentoring process, training, matching, oversight by the host organization or the frequency, and quality of interactions as well as differences in mentor-mentee ratios.	Sng et al^15^; Tan et al^16^; Ikbal et al^57^; Qiao Ting Low et al^58^
Relational-dependent	Mentoring processes “pivot on how mentor and mentee interact in different settings over time and in the face of different pressures and goals,” and “appears to be a function of [their] compatibility.”	Ikbal et al^57^ Sng et al^15^
	A more robust and stronger relationship can withstand and adapt to difficulties faced. The relationship can be strengthened as mentors and mentees are reciprocally empowered with skills, knowledge and confidence.	Ikbal et al^57^
	For this to occur, mentors and mentees must “[remain] motivated and invested in the shared goals of the mentoring process.”	Sng et al^15^
	The host organization also has a role to play in facilitating the strengthening of the mentoring relationship.	Sng et al^15^
Environment-dependent	Guidelines, such as those set by the host organization, influence how mentoring is carried out by mentors and mentees.	Sng et al^15^
	Oversight of the program such as through the matching process or mentee-mentor interactions, and support rendered also affects how mentoring is carried out.	Sng et al^15^; Ikbal et al^57^
Entwined	As mentioned, mentoring is dependent on the factors listed above. These factors do not impact mentoring independently of each other. Some mentoring programs have failed by neglecting certain factors in favor for others.	Sng et al^15^; Ikbal et al^57^

### Stakeholders

By virtue of their intrinsic involvement, stakeholders also naturally influence the mentoring process and their outcomes. This underscores the gravity of the matching process in ensuring that would-be mentees and mentors possess desirable traits that complement one another and the overall goals of the program.^57,58^ These mentee- and mentor-specific traits are delineated in Supplemental Appendices 4 and 5 but may be broadly categorized into personal and professional characteristics.

In addition, their ability to fulfil their respective roles and responsibilities foregrounds the importance of longitudinal training, assessment and support.^15,16,17^ Indeed, the host organization, oft forgotten as a pivotal stakeholder, plays a crucial role in overseeing, supporting and assessing various facets of the mentoring program—including mentor training, mentee briefing, the matching process, mentoring relationship, mentoring approach, and mentoring environment.^15,57,58,60^

### Mentoring relationship

Qiao Ting Low et al^58^ argue that mentoring relationships are shaped by shared values and beliefs between mentees and mentors, their success hinged on the presence of quality and reciprocal interactions between all stakeholders, including the host organization.^57,58^ The mentoring relationship has notable bearings on the professional identity formation of mentees and the motivations of mentors in seeking out avenues to groom them. Indeed the success of the overall mentoring program in realizing its mentoring goals is said to pivot upon the nurturing of lasting and personalized mentoring relationships.^60^

### Mentoring approach

Most mentoring programs adopt either a formal or informal mentoring approach. Formal mentoring offers a more rigorous structure to the matching process and provides clearer specifications with regards to goals, learning objectives, roles and responsibilities, codes of conduct, standards of practice, and the type and duration of interactions expected.^60^

Conversely, informal mentoring “*revolves around the idea of apprenticeship in medicine*”^57^ and sees more ad-hoc influence by learners, tutors, and the host organization.^60^ Whilst it proffers a more collegial environment which facilitates open communication beneficial toward the development of stronger ties between mentees and mentors, the lack of protected time and poor oversight, transparency, and support from the host threaten the viability of informal mentoring processes.

Indeed regardless of which mentoring approach is used, for its full efficacy it should be sensitive to the personal, academic, research, professional, social, and emotional considerations of individual stakeholders involved. This is paramount as mentee and mentor knowledge, experience, preferences, needs, and limitations affect their availability and commitment toward developing the mentoring relationship. Whilst acceptable practice parameters must be adhered to, the importance of a flexible mentoring approach is underpinned here.

### Mentoring environment

Hee et al^60^ suggest that the mentoring environment has 2 interwoven features, the mentoring structure and mentoring culture.

The mentoring structure is defined as “*the framework that shapes the learning approach.*” It includes logistical considerations such as the provision of protected time; the location, frequency, and duration of mentoring, tutorial, and feedback sessions; and the presence of confidential avenues for raising concerns directly to the host organization. The mentoring structure lays the foundation for the provision of streamlined professional and personal support to mentees and mentors alike.

The mentoring culture on the other hand refers to “*the norms, values, beliefs, practices and support moulding the socioemotional environment in which learning occurs.*” These include permissible topics and sanctioned behaviors during the mentoring process. It is informed by the stakeholders, the formal mentoring structure and the informal curriculum. The latter referring to opportunistic and idiosyncratic instruction-giving, including the transmission of values and beliefs that underlie prevailing actions and practices of mentors and the host.

### Benefits of mentoring

The diverse benefits of mentoring to mentees are highlighted in Table 3.

**Table 3.** Benefits of mentoring to mentees.

Benefits	References
Personal	
Personal development	Ikbal et al^57^; Qiao Ting Low et al^58^; Tohet al^59^
- Increased sense of self-efficacy and self-confidence - Increased psychological and behavioral competence - Career/fellowship - Mentoring program - Career mentoring advice - Elective advice	
Professional	
Professional abilities	Ikbal et al^57^; Qiao Ting Low et al^58^; Tohet al^59^
- Increased sense of self-efficacy and self-confidence - Improved communication skills - Expansion and consolidation of social skills - Emotional and psychological support	
Career	
- Developing professional identities - Career guidance, support, and advice - Opportunities for career advancement - Enhanced job satisfaction - Influence on career path - Residency application process	
Clinical	
- Improved clinical and interpersonal skills - Improved patient care	
Academic (research)	
- Increased research productivity - Improved research skills - Better research opportunities - Improved support and resources for research - Improved research time allocation	
Academic (non-research)	
- Becoming a self-directed learner - Improved teaching skills - Increased professional society and committee nominations	
Others	
- Receives guidance in time management allowing for better quality of life - Improved medical school performance - Improved institutional support and backing	

Although not commonly addressed, benefits to mentors may be personal and or professional. Personal benefits include the chance to share their knowledge and experience leading to satisfaction, joy, fulfilment, and pride when witnessing the success of their mentees. It also reportedly encourages professional growth, improved job performance, accelerated research productivity and promotions, and provides opportunities to forge new liaisons with collaborators.^15,57,58,59,60^

### Barriers to effective mentoring

Barriers to effective mentoring include a lack of protected time, availability of mentors and difficulties in balancing disparate needs, goals, and expectations of the stakeholders involved.^16^ These hinder the fostering of fruitful mentee-mentor relationships.

### Assessment in mentoring

Traditionally under the purview of the host organization, assessments and evaluations of the mentoring process remain poorly studied.^15,16,59,60^ No validated tool to assess mentoring experiences or their outcomes as presently available.

### Mentoring theories

Despite recent efforts to forward a theory of mentoring,^56,61^ there are no prevailing theories that comprehensively capture all its components.

### Stage 5: Synthesis of the narrative

The narrative produced was guided by the Best Evidence Medical Education (BEME) Collaboration guide^62^ and the STORIES (Structured approach to the Reporting In healthcare education of Evidence Synthesis) statement. In addressing its primary and secondary research questions, this NR in SEBA builds on the 10 themes/categories identified to map and discuss prevailing data on novice mentoring.

#### Definitions of novice mentoring

With the new findings, novice mentoring may be characterized as the process of creating personalized, enduring, and mutually beneficial mentoring relationships between stakeholders. Its success necessitates the guidance of a mentoring structure that is able to balance demands for flexibility in accommodating for evolving mentoring needs, goals, circumstances of stakeholders, and the mentoring environment *yet* maintain a consistent mentoring approach that exists within the confines of codes, standards of practice, and program expectations.

To support novice mentoring’s dynamic, entwined, evolving, adaptable, context-specific, goal-sensitive, mentee-, mentor-, host organization-, mentoring environment-, mentoring approach-, and mentoring relationship-dependent nature, the host organization must oversee, assess, and support the mentees, mentors, matching process, mentoring relationship, mentoring approach, and mentoring environment.

This definition will help to focus the course and content of this NR in SEBA.

#### Role of host organization

Recent reviews suggest that the host organization plays a critical role in ensuring consistency^3,15,59,63-88^ yet flexibility^15,16,45,59,67,69-71,73-79,81,84,89-103^ within the mentoring program. By spearheading the program, the host organization is deeply involved in formalizing the mentoring approach and its structure. It establishes mentee and mentor roles, responsibilities, and expectations;^82,83,104^ practice standards and codes of conduct;^15,59,66,69,75,76,105^ and the milestones of each mentoring stage.^85,96^ Albeit under the aegis of the mentoring program as a whole, guidelines established by the host steer mentees and mentors as they set their specific goals,^15,45,59,61,63,76,96,106,107^ objectives,^15,87,96,98,106^ and timelines.

Whilst a consistent and transparent program imbues confidence in the mentoring relationship, the mentee’s and mentor’s sense of autonomy, connectivity, and advocacy of the program^88^ is enhanced by flexible accommodations to their particular setting, goals, needs, and capabilities. Understanding the host organization’s role in the mentoring process and in operationalizing the mentoring structure it has adopted underlines the influence of the mentoring culture and introduces the notion of mentoring dynamics.

#### Mentoring culture and dynamics

Mentoring dynamics refer to the quality of interactions between stakeholders. It is critical to the development of mentoring relationships is influenced by particularly influenced by the mentoring culture^59,81,83,84,108^ which enables the host to instil and influence mentoring and education philosophies as well as goals and values of the program.^15,67,74,77,80,81,92,109^ The mentoring culture consequences recruitment and retention of stakeholders^3,63,65,67,71,76,81,90,92,98,110-112^ and informs stakeholders of characteristics desired of them.

#### The mentoring ecosystem

The notion that each stakeholder influences the mentoring dynamic raises the idea of a mentoring ecosystem with each stakeholder bringing with them individual “micro-environment.” For mentees and mentors, their micro-environment consists of internal and external factors. Internal factors account for individual characteristics, availabilities, abilities, motivations, and goals. These are impacted by external factors such as particular sociocultural, curricular, personal, academic, clinical, professional, ethical, and research factors; prevailing geopolitical, care and educational financing as well as healthcare and educational systems. Changes in these factors affect their micro-environment and influences their ability to productively participate in the mentoring process.

The host organization’s microenvironment is more complex. Despite nurturing the program’s mentoring environment, the host organization’s micro-environment does not on its own shape it. Instead the host organization’s “micro-environment” which is informed by internal and external factors including the mentoring structure, the nature and dynamics of interactions between stakeholders and the informal, formal and hidden curricula shape the program’s own mentoring environment.

The mentee’s and mentor’s micro-environment are influenced by the program’s mentoring environment as early as the recruitment stage where participation is contemplated. At the matching stage where mentees are introduced to potential mentors, their micro-environment begin to intermingle. Within this “meso-environment,” elements within the mentee’s micro-environment affect the mentor’s ability to function within the mentoring program and vice versa.

The mentoring relationship begins with the mentee’s and mentor’s formal agreement to enter into a mentoring relationship with each other under the aegis of the mentoring program. With this, the mentee’s and mentor’s meso-environment fuse with the host’s microenvironment as well as the program’s mentoring environment to form the macro-environment.

This macro-environment sees the mentor and mentee influenced by wider factors affecting the mentoring program and host organization. The macro-environment will change as the stakeholders’ micro-environment interact with one another and as the mentoring relationship moves through the mentoring stages. Thus the evolution of the mentoring micro-, meso-, and macro-environments is directed and heavily influenced by the overarching mentoring structure and its constituents. This gives rise to the idea of a mentoring ecosystem which comprises of these dynamic elements and interactions. How micro-, meso-, and macro-environments form under the aegis of the mentoring structure also uncovers the novel role of competency based mentoring stages.

#### Competency based mentoring stages

The more traditional view of mentoring relationships is that it begins with and is sustained by a “fit for purpose” match that brings mentees and mentors with complementary interests and goals together. A newer perspective however sees the development of mentoring relationships occur in stages. To create a stable, effective and nurturing environment, Krishna et al^17^ posit clear mentoring stages in the form of recruitment; aligning of expectations; the matching process; pre-mentoring meetings and training processes; and the subsequent mentoring relationship. For more targeted support and efficacious mentoring outcomes, it was suggested that each stage contain specific competencies to be met by the mentees in order for the mentoring relationship to advance. Not only do these competency based stages shed light as to how micro-, meso-, and macro-environments interact and respond to rigorous mentoring structures but it also further explains the importance of balancing between such need for consistency and demands for flexibility.

**Figure 2.**
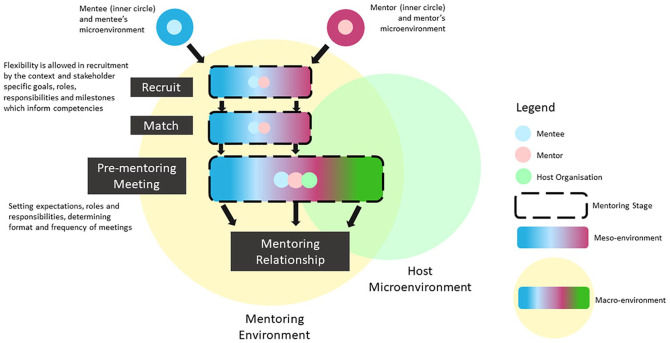
The mentoring ecosystem.

In order to conceptualize these dynamic elements and interactions within the mentoring ecosystem, Figure 2 was drawn up. Here, stages are delineated as boxes linked by arrows suggesting that progression along the mentoring stages are usually fixed and unidirectional. The borders of these boxes represent codes of practice, education and professional standards, roles, responsibilities, and milestones which collectively inform the “competencies” required at the specific stage. Having the competencies as part of the box underlines the inherent variability that is present within each mentoring relationship. The boxes are also delineated as broken lines to suggest that the developing mentoring relationship may be influenced by the wider mentoring environment and its constituent micro-, meso-, and macro-environments and mentoring structure. Yet these borders do not allow adaptations to accommodate to stakeholder needs, goals, and practices if they breach the confines of the specific stage’s acceptable practice parameters. This encapsulates how a base-line standard of consistency may be rigorously introduced to the mentoring process.

#### Proposed mentoring framework

As PC programs wrangle with concerns about structural issues in mentoring programs that may predispose ethical issues and abuse, we forward a mentoring framework built around the mentoring stages posited by Krishna et al^17^ (Table 4). Advancing a mentoring structure that revolves around a set of core competencies allows for flexibility to contend with changes in the stakeholder’s microenvironments and the mentoring relationship’s macro-environment whilst ensuring that minimum standards are met. In addition, having specific competencies will greatly streamline expectations and guide single-stop and longitudinal assessments which are presently lacking in the literature. The associated milestones may help to identify at-risk relationships and allow for remediation and personalized, appropriate, specific and timely support to be provided to struggling mentees and mentors by the host organization.

**Table 4.** Proposed mentoring framework delineated through mentoring stages.

Stages	Requirements for this stage	Competencies to be achieved before progression to next stage
Recruitment	Host organization • Carries out a needs assessment and determines the role and goals of the mentoring program • Establishes the mentoring goals, outcomes, timelines and mentoring approach and the matching, assessment and support mechanism to be employed by the program • Based on the program goals, recruits interested and suitable mentors and mentees • Host organization assess mentee’s and mentor’s suitability for program. • Host organization to briefs would be mentees and mentors on the program and helps align expectations	Mentees and mentors • Determine if the mentoring goals, outcomes, timelines and mentoring approach and the matching, assessment, and support mechanism is suitable for their individual needs • Recognize, evaluate, and indicate own interest in mentoring program • Align expectations with the program goals and outcomes Host organization • Identify and recruit suitable mentors and mentees • Organize briefings for would be mentors and mentees and align expectations
Matching	Host organization • Determine the desirable characteristics of mentees and mentors • Determine the skills sets and levels of knowledge and experience required of would be mentees and mentors • Evaluate mentors and mentees upon their personal and professional characteristics, goals, abilities, interests and complementary practices and traits • Introduce mentees to potential mentors based upon the aforementioned factors	Mentees and mentors • Reflection and make an honest assessment of their own personal and professional characteristics, goals, abilities, interests and desired practices and traits in their mentoring partners • Communicate the aforementioned factors to the host organization for matching Host organization • Determine the personal and professional characteristics, goals, abilities, interests and complementary practices and traits required of would be mentees and mentors and infuse these into the “criterion based” matching process • Identification of suitable matches
Pre-mentoring meeting	Mentees and mentors • Would be mentoring pairs meet to discuss their interests and goals, determine viable timelines and establish responsibilities, roles, codes of conduct, outcome measures, assessment methods, and support mechanism	Mentees and mentors • Alignment of expectations • Agreement on responsibilities, roles, codes of conduct, outcome measures, assessment methods, and support mechanism that may be have been customized/adapted by mentees and mentors • Mentees and mentors make a decision if they would like to proceed to formalize their mentoring relationship Host organization • Provide a platform for pre-mentoring meeting • Establish a set of responsibilities, roles, codes of conduct, outcome measures, assessment methods, and support mechanism that may be further customized/adapted by mentees and mentors
Mentoring relationship	Mentees and mentors • Mentors and mentees meet as per guidelines set by the host organization and at a frequency and location/medium as agreed upon by both parties. • Mentors provide personalized support for mentees with open communication between both parties. • Mentors and mentees work towards fulfilment of previously agreed upon goals Host organization • Monitoring of mentoring relationships longitudinally such as through assessments • Provide longitudinal mentor and mentee training	Mentees and mentors • Meet the objectives of each sub-stage of the mentoring process • Provide feedback • Ask for help early and clearly Host organization • Assess the mentoring relationship • Police codes of practice • Direct support in a timely and appropriate manner

## Limitations

Whilst NRs attempt to build on linked papers, the lack of data on novice mentoring and the common root from which they stem from hinders efforts to create a “coherent paradigm.”^38^ In addition although use of Levac et al’s^113^ adaptation of Arksey and O’Malley’s^114^ methodological framework to conduct a systematic scoping review rather than “traditional” scoping reviews^115^ was used to enhance transparency and reproducibility of the NR and use of broad search terms, the presence of papers drawn from a common stable raises concerns about inherent biases and the omission of critical papers. These factors stifle efforts to deepen understanding of a complex process^116^ despite use of the Split Approach and involvement of expert teams. However, the expert team concurred on the narrative presented and believed that it would be useful for various parties interested in designing, assessing, supporting, and expanding novice mentoring programs.

## Conclusion

Conceiving mentoring as a flexible process within structured mentoring stages helps explain the process of balance and underlines the ability of the mentoring structure to personalize mentoring processes and contend with evolving mentoring dynamics across different stages without compromising mentoring standards and breaching codes of practice. Perhaps more importantly having the competencies and the standards of practice of the specific stage agreed upon by the stakeholders makes for more timely, personalized, appropriate, specific assessments of the mentoring dynamics, relationship, and progress. Moving forward, future studies must also consider the impact of PC’s multidisciplinary practice and the impact of multiple stakeholders in the mentoring process and enhance the structuring of the mentoring process accordingly. Assessments of mentoring processes and mentor training must be the focus of coming studies if mentoring in PC is to regain its place in PC education.

## Supplemental Material

Appendices_xyz46891a36bb924 – Supplemental material for Enhancing Mentoring in Palliative Care: An Evidence Based Mentoring Framework Click here for additional data file. Supplemental material, Appendices_xyz46891a36bb924 for Enhancing Mentoring in Palliative Care: An Evidence Based Mentoring Framework by Lalit Kumar Radha Krishna, Lorraine Hui En Tan, Yun Ting Ong, Kuang Teck Tay, Jia Min Hee, Min Chiam, Elisha Wan Ying Chia, Krish Sheri, Xiu Hui Tan, Yao Hao Teo, Cheryl Shumin Kow, Stephen Mason and Ying Pin Toh in Journal of Medical Education and Curricular Development
