# Supplementary material for: Enhancing Mentoring in Palliative Care: An Evidence Based Mentoring Framework
Source: J Med Educ Curric Dev. 2020 Sep 23;7:2382120520957649. doi: 10.1177/2382120520957649 (PMC7517982; doi:10.1177/2382120520957649)
Supplement: Appendices_xyz46891a36bb924 – Supplemental material for Enhancing Mentoring in Palliative Care: An Evidence Based Mentoring Framework [file Appendices_xyz46891a36bb924.pdf]

Appendices

Appendix 1: PubMed Search Strategy

(mentoring[MeSH] OR mentors[MeSH] OR mentor[tiab] OR mentors[tiab] OR mentoring[tiab] OR mentorship[tiab] OR “mentoring structure”[tiab] OR “mentoring structures”[tiab] OR “mentoring relationship”[tiab] OR “mentoring relationships”[tiab] OR “mentoring environment”[tiab] OR “mentoring environments”[tiab]) AND (“Schools, Medical”[MeSH] OR “Medicine”[MeSH] OR “Students, Medical”[MeSH] OR “medical student”[tiab] OR “medical students”[tiab] OR “medical school”[tiab] OR “medical schools”[tiab] OR medicine[tiab])

Appendix 2. Tabulated Summary of Key Findings in the Included Articles

Author, Year	Title	Background	Theoretical Approach and Methods	Main empirical findings	Insights drawn
Sng et al, 2017	Mentoring relationships between senior physicians and junior doctors and/or medical students: A thematic review	Sng et al (2017) identified mentoring relationships as being key to mentoring success.	Not explicitly stated though it would appear that this thematic review was built on a constructivist approach and a relativist lens combining a wide range of socially constructed accounts of mentoring experiences from the perspective of mentees and mentors.<br><br>Employed Braun and Clarke's approach to thematic analysis	18,915 articles retrieved, of which 329 full text articles were independently evaluated by the three authors and 49 articles were included. 5 themes were identified including<br><br>1) Initiation Process, - includes goal setting, mentor training, mentee briefing, desired characteristics of mentees and mentors and initiation of relationships and the matching process<br>2) Developmental Process- includes relational characteristics<br>3) Evaluation Process,<br>4) Sustaining mentoring relationship,<br>5) Obstacles to Effective Mentoring	● The impact of the mentoring environment<br>● Mentoring's entwined, personalized, context-sensitive, goal-specific, relational, mentee, mentor and host-organization dependent<br>● Highlighted the need for design of longitudinal mentoring tools, the need to better appreciate the role of the host organization, the matching process and the mentoring environment and the need for a theory of mentoring to guide the mentoring process.
Tan et al., 2018	A framework for mentoring of medical students: thematic analysis of mentoring programmes between 2000 and 2015	Tan et al (2018) seeks to a consistent, transparent and efficient approach to novice mentoring will ensure effective oversight of mentoring processes and	Not explicitly stated though it would appear that this thematic review was built on a constructivist approach and a relativist lens	25,605 abstracts were retrieved, 162 full-text articles were reviewed and 34 articles were included. Thematic analysis of the 6 cross sectional studies, 27	● The Mentoring Framework pivots upon 5 pillars: (a) programmatic structure; (b) over- sight by a host organization; (c) integrating mentoring with existing medical training

		safeguard against the potential for abuse of mentoring relationships	combining a wide range of socially constructed accounts of mentoring experiences from the perspective of mentees and mentors.<br><br>Employed Braun and Clarke's approach to thematic analysis	case studies and 1 commentary revealed 4 themes:<br><br>1) preparation- mentor training and selection, mentee preparation, structured program including mentor to mentee ratios which considers one to one, group and mixed mentoring and mentoring culture<br>2) initiating the mentoring process- types of matching,<br>3) supporting the mentoring process – mentoring resources, incentives, financial and administrative support<br>4) the obstacles to effective mentoring- lack of time, faculty recruitment and the need to balance flexibility and structure	curricula; (d) employing a guided matching process; (e) recommendation for mentor and mentee training.
Hee et al, 2018	Understanding the Mentoring Environment through Thematic Analysis of the Learning Environment in Medicine	Hee et al (2018) seek to better understand the mentoring environment (ME) through the study of the closely related and better studied concept learning culture. The authors suggest that better understanding of the ME will allow better nurturing of mentoring programs, relationships and processes.	Not explicitly stated though it would appear that this thematic review was built on a constructivist approach and a relativist lens combining a wide range of socially constructed accounts of mentoring experiences from the perspective of mentees and mentors.	4574 abstracts, reviewed 90 full-text articles and included 58 articles in this review that revealed two themes<br><br>1) mentoring structure – formal curriculum, host organization, mentee, mentor, the mentoring relationship.<br>2) mentoring culture- informal and hidden curriculum, host organization, mentee, mentor, the mentoring relationship.	• Mentoring relationships consist of the mentee, mentor, mentoring relationship, the wider healthcare and education systems (elements). Each element is interdependent and brings with it, its own goals, timelines, practices, code of conduct and expectations that creates a structure and culture to the ME. The influence and co-dependence of elements within the ME culture and the ME structure underline the "entwined, evolving, adapting,

			Employed Braun and Clarke's approach to thematic analysis		context-specific, goal-sensitive, learner-, tutor-, relationship- and host organization-dependent nature" of the ME (henceforth ME's nature). MEs can be seen as being "shaped by the nature, culture and structure of evolving mentoring relationships between the mentor, the mentee, the host organization and the curriculum (henceforth quartet of stakeholders). MEs evolve to support and nurture mentoring relationships in dynamic conditions and as particular relationships amongst the quartet of stakeholders change. MEs influence the quality of education and professional practice".
Low et al, 2018	A narrative review of mentoring programmes in general practice, Education for Primary Care	Low et al (2018) seek to better understand the mentoring process and relationship in general practice in order to better structure, support and oversee the processes.	Not explicitly stated though it would appear that this thematic review was built on a constructivist approach and a relativist lens combining a wide range of socially constructed accounts of mentoring experiences from the perspective of mentees and mentors.	1973 abstracts, reviewed 20 full-text articles and included 9 articles in this review that revealed five themes including characteristics of the<br><br>1) mentoring process, -initiating, mentee-to-mentor, preliminary meetings, mentoring approaches,<br>2) mentoring relationship,<br>3) mentors-characteristics, roles,	● Initiation of mentoring relationships should be overseen and facilitated by the host organization which must support the matching, training and oversight of the mentoring process, mentees should be given a choice to select from a list of approved trained mentors, supported by a pre-mentoring process<br>● Mentees and mentors must be supported and acknowledged for their contributions

			Employed Braun and Clarke's approach to thematic analysis	responsibilities, benefits and challenges for mentors<br>4) mentee- benefits of mentoring for mentees<br>5) organization and stakeholders.	• The role of ME is highlighted but not elaborated due to a lack of data
Toh et al, 2017	Developing Palliative Care Physicians through Mentoring Relationships.	Mentoring is widely used however the longterm effects of mentoring is poorly understood. Toh et al (2017) carry out a literature review of "the perspectives of mentors and mentees featured in editorials, perspective and opinion pieces to provide long-term views and reflections of mentoring experiences so lacking in mentoring in medicine"	Not explicitly stated though it would appear that this thematic review was built on a constructivist approach and a relativist lens combining a wide range of socially constructed accounts of mentoring experiences from the perspective of mentees and mentors.<br><br>Employed Braun and Clarke's approach to thematic analysis	806 abstracts, reviewed 63 full-text articles and included 39 articles in this review that revealed 4 themes including characteristics of<br><br>1) the mentoring relationship- safe space, empowerment, mutually beneficial<br>2) the role of the mentor<br>3) the characteristics of the mentee<br>4) and advice to mentees and mentors in advancing effective mentoring relationship	• Evidence of longterm effects necessitate effective oversight, assessments and structure to the mentoring process.
Ikbal et al, 2017	Mentoring in Palliative Medicine. A thematic analysis of mentoring in Internal medicine between 2000 and 2015	Ikbal et al (2017) seek to identify key elements of successful mentoring programs.	Not explicitly stated though it would appear that this thematic review was built on a constructivist approach and a relativist lens combining a wide range of socially constructed accounts of mentoring experiences	466 abstracts, reviewed 17 full-text articles and included 7 articles in this review that revealed 8 themes including<br><br>1) definition of mentoring<br>2) approach, - initiating, mentee-to-mentor, preliminary meetings, mentoring approaches,<br>3) characteristics mentoring relationship- built of common values, reciprocal and evolving relationship	• Delineation of mentoring's nature, highlights the importance of the mentoring environment and the host organization and due consideration of multidisciplinary mentoring

			from the perspective of mentees and mentors.<br><br>Employed Braun and Clarke's approach to thematic analysis	4) mentoring program-mentor training, matching, initiation and duration setting<br>5) desired characteristics of mentor<br>6) desired characteristics of mentee<br>7) benefits and drawbacks of mentoring	
Loo et al., 2017	Towards a Practice Guided Evidence Based Theory of Mentoring in Palliative Care	Maintains that without a mentoring theory, mentoring cannot take its role in medical education	An opinion piece considering articles by Wu et al and Wahab et al on mentoring in PM.	1) Having reviewed the Adult Learning Theory and the Cognitive learning theory – the authors propose Krishna's Mentoring Pyramid as a means of understanding mentoring.	• Based on the Krishna's Mentoring Pyramid, the authors adopt Hamdy and Taylor's multitheories approach to combine Kolb's experiential learning, Adult Learning Theory and the Cognitive learning theory to suggest the basis for a mentoring theory
Yeam et al, 2016	An Evidence-Based Evaluation of Prevailing Learning Theories on Mentoring in Palliative Medicine.	Yeam et al (2016) seek to identify "a learning theory of mentoring in Palliative Medicine is necessary to guide the development of an effective mentoring program in this speciality"	Not explicitly stated though it would appear that this is an opinion paper built upon a literature review<br><br>Thematic analysis was adopted	The PRISMA was not provided but it would 6 reviews were evaluated<br><br>1) Krishna's mentoring Model- reveals<br>a. Mentor dependent factors<br>b. Mentee dependent factors<br>c. Organizational dependent factors<br>d. Goals specific<br>e. Context sensitive<br>f. The evolving nature of mentoring relationships<br>g. The quality of mentoring relationships<br>h. Mentoring environment	• Mentoring theories do not fully capture all the aspects of Krishna's model of mentoring

				2) the apprenticeship model- focuses upon "being a PM rather than learning about PM". It is dependent upon the mentor skills<br>3) Multi-theories approach – focuses upon the 5 stages of the mentoring relationship- which are highly theoretical<br>4) Multiple mentors theory- depends on the mentoring relationship which depends on the network. The relationships within the network, the diversity of the network and the strength of the relationships	
Krishna, Toh, Mason, Kanesvaran, 2019	Mentoring stages: A study of undergraduate mentoring in palliative medicine in Singapore	Mentoring nurtures a mentee's personal and professional development. Yet conflation of mentoring approaches and a failure to contend with mentoring's nature makes it difficult to study mentoring processes and relationships. This study aims to understand of mentee experiences in the Palliative Medicine Initiative (PMI).	Not explicitly stated though it would appear that this review was built on a constructivist approach and a relativist lens combining a wide range of socially constructed accounts of mentoring experiences from the perspective of mentees and mentors.<br><br>A qualitative approach via semi-structured interviews was adopted. The authors also employed Braun	Two themes were identified from thematic analysis:<br><br>1) Stages of mentoring – this outlines the 6 stages of mentoring: pre-mentoring, initial research meetings, data gathering, review of study findings, manuscript preparation and reflections and aspirations<br>2) Communication – this includes the characteristics of mentors that influenced communication, the nature of the mentor-mentee interactions, and the barriers to mentor-mentee interactions	● Each stage of the mentoring process cascades into the next, forming the basis for the stages that follow<br>● Mentoring stages may be viewed as 'circumscribed sequential projects' with 'specific goals and competency requirements' that build on one another to achieve an overarching goal. Each stage requires the mentee to attain specific skills, insights and abilities, making progress from one mentoring stage to the next, highlighting a competency-based process<br>● There is a need to need to adapt its mentoring approach to accommodate to diverse influences upon the mentoring process without

			and Clarke's approach to thematic analysis.		compromising mentoring experiences and breaching prevailing standards of practice and codes of conduct.<br>● Adaptability within the mentoring approach is evident at all mentoring stages.
Krishna et al, 2019	Educational roles as a continuum of mentoring's role in medicine – a systematic review and thematic analysis of educational studies from 2000 to 2018	The author aims to evaluate the notion that teaching, tutoring, role modelling, coaching and supervision may all be part of the mentoring process.	Not explicitly stated though it would appear that systematic reviews stem from a positivist approach.<br><br>The authors employed Arksey and O'Malley (2005)'s and Levac et al (2010)'s approach to systematic review. They also used Braun and Clarke's approach to thematic analysis.	Thematic analysis of the prevailing descriptions of role modelling, teaching, tutoring, coaching and supervision were carried out. Their characteristics and descriptions are highlighted.<br><br>1) Educational processes - are interactive, context-specific, goal-sensitive and dynamic process that are guided by the objectives of the clinical training program and supported and overseen by a host organization.<br>2) Nature of relationship – this includes role modelling, teaching and tutoring, coaching and supervision.<br>3) Problems faced in four educational roles – common issues include insufficient training, poor program structure, inadequate learning resources and inaccurate program evaluation and learning assessment.	● The mentoring spectrum describes a range of educa- tional practices contained under the aegis of mentoring be- ginning with role modelling on the left side of the spectrum and mentoring on the right.<br>● Implications for how mentoring spectrum should be applied – mentors need to be trained in a range of educational approaches, there is a need for clear standards of practice, codes of conduct and practice guidelines, and for mentee-mentor expectations to be aligned, the matching process must be appropriate<br>● Enablers include mentors that are trained and mentored, briefed on CoPs, expectations and responsibilities, assessments, support from host organization, well-structured and safe and open learning environment.
Wu et al, 2016	Toward an Interprofessional Mentoring Program in	The author aims to analyze mentoring programs in medicine, surgery, nursing	Grounded Theory was employed to	A total of 1059 abstracts were retrieved and evaluated, 61 full-text reviews were analyzed and	● Success of mentorship programs hinge upon the context sensitive,

	Palliative Care - A Review of Undergraduate and Postgraduate Mentoring in Medicine, Nursing, Surgery and Social Work	and social work in order to identify key elements and common facets of successful mentoring programs that can be used to create a multi-professional mentoring program in Palliative Care.<br><br>(Note: only elements of mentoring relevant to Internal Medicine will be reported in this summary)	thematically analyze the review articles.	20 reviews were included. The following themes were revealed:<br><br>1) Definition of mentoring – there is complexity and diversity of understanding and practice of mentoring<br>2) Formal mentoring in medicine – suggests an increase mentoring pairs and improve inclusion of minority groups; officially sanctioned mentoring relationships are more likely to persist<br>3) Informal mentoring in medicine – recommends mentees select their own mentors; informal mentoring facilitates emotional security, honest reflection and reduces fears of failure.<br>4) Considerations when choosing appropriate form of mentoring relationships – should consider a wide range of issues<br>5) Mentoring approaches – this includes dyadic mentoring, group mentoring, mosaic mentoring, mentoring via a combination of methods, e-mentoring and distance mentoring<br>6) Elements of the mentoring process – this includes mentor training, initiation of mentoring, mentoring objectives, duration and frequency of mentor	goal specific and mentee- and mentor- dependent features.<br>● Undergraduate mentoring goals tend to focus on professional development whilst postgraduate mentoring tends to require more holistic mentoring support.<br>● Recommendations for a Palliative Care mentoring program include – adopt a formal process, supported by host organizations, allow mentees to select mentors, adopt mosaic mentoring supported by e-mentoring.

				meetings and the impact of race, ethnicity and gender.	
Wahab et al, 2016	Creating Effective Interprofessional Mentoring Relationships in Palliative Care- Lessons from Medicine, Nursing, Surgery and Social Work	Mentoring is seen as an effective means of facilitating multi-professional collaborations however little data exists on operationalizing an interprofessional mentoring program in Palliative Care. To address this gap and circumnavigate the context-specific nature of mentoring, the authors scrutinized mentoring approaches in medicine, surgery, nursing and medical social work to identify common elements of mentoring within their respective practices that will provide the basis of an interprofessional mentoring in Palliative Care.<br><br>(Note: only elements of mentoring relevant to Internal Medicine will be reported in this summary)	Grounded Theory was employed to thematically analyse the review articles.	A total of 1059 abstracts were retrieved and evaluated, 61 full-text articles were analyzed and 20 reviews were included in this review. 6 themes were revealed:<br><br>1) Characteristics of prevailing definitions of mentoring – interpersonal, dynamic, beneficial relationship, shared interests<br>2) Characteristics of mentor in medicine – personal, professional and undesirable characteristics<br>3) Characteristics of mentee in medicine – personal, professional and undesirable characteristics<br>4) Benefits to mentoring – benefits to mentee and mentor in the domains of personal and professional life, benefits to shareholders (improved patients outcomes, employee turnovers, increased institutional stability)<br>5) Drawbacks of mentoring – this includes mentoring abuse, and a lack of recognition in mentors	Recommendations for an IPE-based mentoring program in Palliative Care include:<br><br>● The impact of mentors, mentees and host organization are important factors that underlie the unique interpersonal relationship in the mentoring program. Efforts should continue to facilitate interpersonal relationships.<br>● Mentoring relationships are dynamic and its ultimate goal should be for the friendship to blossom and the development of a collegial relationship between peers.<br>● Host organizations play a crucial role in mentoring, by supporting and facilitating mentoring relationships.
Tan et al, 2017	Extending Mentoring in Palliative Medicine -	The authors seeks to advance a clinically-	Not explicitly stated though it would appear	A total of 1456 citations were reviewed, 133 full text articles	● The social approach to NPG mentoring is

	Systematic Review on Peer, Near-Peer and Group Mentoring in General Medicine	relevant understanding of near peer, peer and group (NPG) mentoring that will help delineate the practice of NPG mentoring and potentially see it blended with novice mentoring.	that systematic reviews stem from a positivist approach.	were retrieved and 8 were included. Themes included:<br><br>1) Structure of NPG mentoring – A blend of informal interactions within a structured process is crucial to its success.<br>2) Benefits of peer, near peer and group mentoring – this is manifold and includes: friendships and collaborations, overcoming disparities in females and ethnic minorities, networking, academic advancement and improving research skills.<br>3) Overcoming common challenges – this includes dissonance in descriptions of practice, conflation of peer, near-peer and group mentoring, determination as to how mentors were selected, the presence of disparate levels of commitment and expectations on the part of the peers and near peers and a lack of clarity on their goals and responsibilities.	strengthened by a basic yet flexible structure that draws curriculum and program designers struggling to imbue their projects with holistic support for learners to NPG mentoring.<br>● NPG mentoring is ideal to supplement novice mentoring to provide timely, appropriate, contextualized, individualized and holistic support.<br>● NPG mentoring is its ability to protect against abuse of the mentoring relationships, which is a growing concern in novice mentoring.<br>● The combination of NPG and novice mentoring also offers an effective means of multidisciplinary mentoring in Palliative Medicine, Pediatrics and Geriatrics. It also raises the possibility of mentoring by near peers from different specialties supporting learners from diverse clinical backgrounds, thus enhancing interprofessional understanding.
Davis and Nakamura, 2010	A Proposed Model for an Optimal Mentoring Environment for Medical Residents: A Literature Review	The author aims to develop a model of the optimal mentoring environment for medical residents. The authors propose that such an environment is a function	Not explicitly stated although it would appear that Literature Reviews draw from a constructivist perspective.	A total of 792 citations were reviewed, and a final 20 articles were included. 2 themes were revealed:<br><br>1) Interactional foundations – this includes	● Interactional foundations enable developmental behaviours.<br>● Residents and attendings should have some say in the mentor– protégé pairing and need to have

		of a relationship that rests upon a set of interactional foundations that allow a protégé to capitalize on the strengths of the mentor, and it facilitates behaviors that will enable the protégé to develop and internalize the requisite knowledge, skills, and attitudes (KSAs) as fully as possible.		emotional safety, responsiveness, support, mentee-centeredness, respect, and informality<br>2) Developmental behaviours - The optimal mentoring environment enables a set of developmental behaviours that foster growth and development. 4 developmental behaviours that appeared with a high frequency in the literature: exercising independence, reflecting, synthesizing, and extrapolating.	sufficient opportunities to meet and to work together.<br>● Mentor training is essential to develop an optimal mentoring environment.<br>● Key factors related to the optimal mentoring environment require additional consideration, particularly the frequency, duration, and quality of meetings. These factors should be measured so that they can be assessed with respect to protégé satisfaction and outcomes. Surveys should include demographic variables like gender, age, and ethnicity to evaluate how they affect success or failure in both the outcomes and the creation of the optimal mentoring environment.
Cheong et al, 2019	A systematic scoping review of ethical issues in mentoring in internal medicine, family medicine and academic medicine	Mentoring's role in medical education is threatened by the potential abuse of mentoring relationships. Particularly affected are mentoring relationships between senior clinicians and junior doctors which lie at the heart of mentoring. To better understand and address these concerns, a systematic scoping review into prevailing accounts of ethical issues and	Theoretical approach not explicitly stated although the philosophical principles of scoping reviews align with the interpretivist paradigm.<br><br>Arksey and O'Malley (2005)'s and Levac et al (2010)'s framework of scoping reviews was utilized. Braun and Clarke (2006)'s	3391 abstracts were identified from the initialy search after removal of duplicates, 412 full-text articles were reviewed, 98 articles were included and thematically analysed.<br><br>1) Ethical issues at individual mentor or mentee level – this includes mentor abuse, a lack of motivation and poor collaborative efforts of mentee. Predisposing factors are likely inadequate training and poor recruitment. Solutions	● There is a need to contextualise ethical concerns within the specific clinical setting and the need for holistic and longitudinal view of mentoring that should alert program designers and administrators to lapses in practice.<br>● The matching process, evaluations of mentoring relationships, oversight and structuring of the mentoring process and culture of the program are areas of the

		professional lapses in mentoring was undertaken.	approach to thematic anal- ysis was adopted	include mentor and mentee training.<br>2) Ethnical issues at level of mentoring relationship – this centres around the nature of interactions in the face of power differentials, and poor management of minority groups in medicine. Predisposing factors include failure to identify and address biases and professional boundaries. Possible solutions include training, establishing clear guidelines, punitive measures for misdemeanour and improved assessment of mentoring relationships.<br>3) Ethical issues at level of host organization – this refers to issues with recruitment, vetting, training, matching and supporting mentors, mentees and their mentoring relationships and in setting the direction and tone of the mentoring approach and environment. Predisposing factors include poor institutional support. Possible solutions include increased host organization support in averting ethical issues affecting mentorships.	mentoring process particularly susceptible to ethical lapses.
Sambunjak, Straus and Marusic, 2009	A Systematic Review of Qualitative Research on the Meaning and Characteristics of	The authors aim to explore and summarize the development, perceptions and experiences of the	Not explicitly stated though it would appear that systematic reviews stem from a positivist approach.	A total of 8,487 citations were identified, 114 full text articles were assessed, and 9 articles were selected for review. Themes were:	• Mentoring can be distinguished from other developmental relation- ships such as leadership or coaching by the broadness of functions it offers.

	Mentoring in Academic Medicine	mentoring relationship in academic medicine.		1) Desired characteristics and actions of the mentor and mentee – mentees should take the initiative to initiate the relationship, be committed, be proactive and willing to learn. Mentor characteristics pertain to mentor's personality, interpersonal abilities and professional status.<br>2) Initiation of mentoring relationships - emerging mentees had the responsibility to find a mentor. Initiation can be via formal or informal means.<br>3) Structure of the mentoring relationship - Structure relates to the gender/race/ethnic composition and the number of actors in the mentoring relationship.<br>4) Characteristics of mentoring relationship – Described as a personal connection, with a set of underlying values<br>5) Barriers to mentoring, dysfunctional mentoring and possible solutions – barriers and solutions exist on a personal, relational and structural level.	● Mentoring in academic medicine is perceived as a uniquely encompassing relationship. Considering some of the mentoring actions described (e.g., helping the mentee to clarify feelings, motivating and fostering self-reflection), a high-quality mentoring relationship is characterized by a high level of personal involvement and commitment, is meant to affect not only the professional, but also the personal lives of the mentor and mentee.
Lim et al, 2019	Enhancing geriatric oncology training through a combination of novice mentoring and peer and near-peer mentoring: A thematic	Training in Geriatric Oncology is in crisis, facing increasing demands in the face of a growing population of older adults, a lack of trainers, and the	Not explicitly stated though it would appear that this review was built on a constructivist approach.	3913 citations were identified, 133 full-text articles were reviewed, and fifteen full-text articles were included. Themes include:	● A key feature of the C-NP mentoring process is the concept of 'tiered mentoring' that sees NP mentors and mentors working together to sup- port mentees, the mentoring relationship, the

	analysis of mentoring in medicine between 2000 and 2017	need to adapt training to different settings and trainee needs. A combination of novice mentoring and near-peer and peer mentoring (C-NP mentoring) has been proposed to provide trainees with personalized training and additional support. The author intends to evaluate the possibility of establishing a C-NP mentoring program in geriatric oncology, through extrapolation of data from well- established practices in Internal Medicine programs.	The Best Evidence Medical Education (BEME) collaboration guide and the STORIES (STructured apprOach to the Reporting In healthcare education of Evidence Synthesis) statement were used to develop a narrative from the thematic analysis of selected articles.<br><br>Braun & Clarke (2006)'s approach to thematic analyses and Sambunjak et al. (2010)'s approach of "negotiated consensual validation" were then used to identify the final list of themes.	1) The role of C-NP mentoring – serve as sources of personalized support, and a chance to select female mentors and mentors from minority groups. Aids in career development.<br>2) Mentee recruitment – this involves prerequisites (needs, abilities, experience, career development) that aid in matching, mentee participation, program size and mentee training.<br>3) Mentor recruitment – this is based upon their specific competencies, level of professional development, and seniority or rank. It involves near-peer mentor recruitment and acknowledging the mentor's contribution.<br>4) Mentor training – This includes mentor training programs, peer mentor workshops on manuscript writing processes and instructional methods.<br>5) Matching – this process is either mentee-initiated or via allocation by host organization.<br>6) The pre-mentoring meetings – This focuses on goal setting, agreeing on ground rules and scheduling of meetings<br>7) Outcomes on C-NP mentoring – Outcomes can be	mentoring project, and evaluate progress of the mentoring process.<br>● The mentor and the NP mentor assess and guide the mentoring process, mentoring relationships, and the mentee's needs and situation. The NP mentor and the mentor may assess different aspects of the mentoring process, share their findings, and adapt the mentoring approach in response to their assessments.<br>● Personalisation is an important aspect of the C-NP program which is also apparent in the matching process. It is important in acknowledging the mentee's needs and psychosocial, academic, research, and professional commitments, and allowing the mentee to play a part in the delineation of the frequency and format of mentoring meetings, setting expectations, roles, and responsibilities, and in establishing project timelines. For mentors, personalization is evident in the structuring of mentoring training and longitudinal support of individual mentors.<br>● There is a need for structure, flexibility, and effective over- sight for an

				measured objectively or subjectively<br>8) Obstacles to C-NP mentoring - The primary obstacles to C-NP mentoring include relationship-, mentor-, mentee-related, and practical problems.<br>9) Proposed improvements to C-NP mentoring - Better structuring of the mentoring program, more mentor training program and the use of asynchronous online mentoring can improve C-NP mentoring.	effective C-NP mentoring program.
Kashiwagi, Varkey and Cook, 2013	Mentoring Programs for Physicians in Academic Medicine: A Systematic Review	Mentoring is vital to professional development in the field of medicine, influencing career choice and faculty retention. The authors reviewed mentoring programs for physicians and aimed to identify key components that contribute to these programs' success.	Not explicitly stated though it would appear that systematic reviews stem from a positivist approach.	The initial search yielded 382 citations. Review of titles and abstracts led to retrieval of 54 full-text articles. 16 article were included.<br><br>1) Models of mentoring programs - Seven mentoring models were described in the reviewed articles: dyad, peer, facilitated peer, speed, functional, group, and distance mentoring.<br>2) Program objectives – Program objectives varied widely. The most common global objectives of mentoring programs were (1) professional or career development, (2) academic success, (3) networking and (4) faculty retention.	● Mentoring programs should be structured to meet each faculty's or institution's specific needs.<br>● Participants are typically highly satisfied with mentoring programs. They perceive that mentoring contributes to their career development, especially in the realms of research and education. Further, faculty retention appears to improve in systems with mentoring programs.

				3) Program components – this consisted of mentor preparation, assessing organizational readiness through open forum discussions, and interviewing administrators to determine successful strategies and potential barriers to program development.<br>4) Evaluation and outcomes – most programs gathered data from mentees, fewer gathered data from mentors. Data collection was predominantly by survey.<br>5) Program evaluation methods – Lack of time was identified as a barrier	
Buddeberg-Fischer and Herta, 2006	Formal mentoring programs for medical students and doctors – a review of the Medline literature	Mentoring programs have been implemented as a specific career-advancement tool in the training and further education of various groups in the medical profession. The authors aimed to examine what types of structured mentoring programs exist for doctors as well as for medical students, what short- and long-term goals these projects pursue, and whether statements can be made on the effectiveness	Not explicitly stated although it would appear that Literature Reviews draw from a constructivist perspective.	Out of 162 papers, only 16 papers fulfilled the described inclusion criteria.<br>1) Formal mentoring programs for medical students and for doctors– this included programs catered for mentees, mentors, with short and long term aims, and varying structure and duration<br>2) Evaluation of programs – largely descriptive in terms of great interest in the offering in question, or a high level of satisfaction among participants.	● Mentoring for doctors must be more stage-specific and goal-oriented for the individual mentee, whilst mentoring for students is also effective addressing a group of mentees at the same training stage.<br>● Most of the reported programs either aim to stimulate students' interest in a certain medical specialty, or as a matter of help and support in earning their specialist degree.<br>● Mentoring leads to the expansion and consolidation of the mentees' professional and social skills.

		and efficiency of these programs.		3) Effectiveness and efficiency of the mentoring programs – financial support for the mentees in the form of grants and/or research funds. Effectiveness or efficiency is not reported to be affected by the cost-effectiveness of the program.	
Sambunjak, Straus and Marusic, 2006	Mentoring in Academic Medicine: A Systematic Review	The authors seek to review the evidence about the prevalence of mentorship and its relationship to career development.	Not explicitly stated though it would appear that systematic reviews stem from a positivist approach.	3640 citations were retrieved from the literature search. Review of abstracts led to retrieval of 142 full-text articles for assessment, and 42 articles were identified for inclusion in the study.<br><br>1) Prevalence and Perceived Importance of Mentorship – scores of various studies were reported<br>2) Impact of Mentorship on Personal Development and Career Guidance – positive impact on personal development and career guidance, with higher rates of promotion.<br>3) Impact of Mentorship on Specialty Choice, Academic Career Choice, and Retention - Mentorship was reported to be an influential factor in the selection of specialty. Respondents working in academic medicine rated the importance of the mentor in their career choices higher than respondents working in non-	• There was a perception that women had more difficulty finding mentors than their colleagues who were men.<br><br>• Mentorship was reported to be an important influence on personal development, career guidance, career choice, and productivity.

				academic primary care settings. Faculty retention is higher in those with pre-ceptoring partnerships.<br>4) Impact of Mentorship on Research Development and Productivity – mentees have greater productivity and higher rates of completion of research<br>5) Differences by Sex in the Mentorship Experience - There were some differences by sex among faculty in perception of the impact of mentorship on success.	
Frei, Stamm and Buddeberg-Fischer, 2010	Mentoring programs for medical students - a review of the PubMed literature 2000 - 2008	The authors aimed to use the review as a starting point for planning and implementing a mentoring program for students at Zurich University Medical School, to find out the types of structured mentoring programs for medical students, objectives pursued by these programs and effects of mentoring programs.	Not explicitly stated although it would appear that Literature Reviews draw from a constructivist perspective.	A total of 438 publications were identified and 25 papers met the inclusion criteria. Themes are:<br>1) Mentoring programs for medical students – This includes goals of mentoring program, career counselling, developing professionalism and personal growth, increasing interest in research and academic careers, fostering interest in certain specialities.<br>2) Mentoring models – includes one-to-one mentorships, in two programs, both settings (e.g. one-to-one and group mentoring).<br>3) Effects of mentoring programs - majority of enrolled	● The mentoring relationship is a reciprocal process which supports juniors in their careers; the benefits as far as the mentors are concerned, however, are rarely described.<br>● The responsibility for keeping the mentorship going rests with the mentees, i.e. bottom-up. If juniors prove to be committed, senior staff will approach them to seek their collaboration in research projects.<br>● Three factors are important for effective mentoring programs. Firstly, for students pursuing an academic career, a one- to-one mentorship with an advanced scientist involving the junior in his/her research proves most

				students were more satisfied in terms of access to career mentoring, elective advice for scheduling the senior year, and the residency application process; they valued the ongoing contact with faculty members and experienced better research opportunities than students graduating before the program was implemented.<br>4) Overviews of mentoring for medical students – this includes characteristics of good mentoring relationships.	effective. Secondly, the mentor must serve as both a professional and personal role model. Thirdly, provision of career counseling by mentors leads to juniors' making an earlier choice in terms of specialty and career.
Beech et al, 2013	Mentoring Programs for Underrepresented Minority Faculty in Academic Medical Centers: A Systematic Review of the Literature	Mentoring is critical for career advancement in academic medicine. However, underrepresented minority (URM) faculty often receive less mentoring than their nonminority peers. The authors conducted a comprehensive review of published mentoring programs designed for URM faculty to identify "promising practices."	Not explicitly stated though it would appear that systematic reviews stem from a positivist approach.	The search identified 73 citations. Abstract reviews led to retrieval of 38 full-text articles for assessment; a final 18 articles were selected for review.<br><br>1) Reach – Structural models for the URM faculty mentoring programs varied. Program participation rates varied greatly.<br>2) Effectiveness - Most articles were largely descriptive and provided minimal objective outcomes, but most included some form of program evaluation.<br>3) Adoption and implementation - Although each mentoring program described unique features, universal aspects included offering training opportunities for early-career	● Each institution should have different approaches to mentoring address specific interests and needs. Additionally, unique institutional environments and cultures influence program design and implementation.<br>● Good practices included one-on-one mentoring by an experienced investigator, group-based skill-building seminars, access to pilot grants, and support for conducting pilot studies. Institutional components, including the support of key leaders and an allocation of resources, are important for sustaining these programs.<br>● Conducting comprehensive program evaluation and dissemination

				URM faculty. Programmatic activities were intended to enhance skills in grant and manuscript writing, the development and delivery of scientific presentations, and didactic teaching.<br>4) Maintenance – Majority of programs began via extramural funding, although many also reported some form of institutional support. Several programs explicitly stated that their goal was to transition from time-limited extramural funding to institutional support.	of findings will be crucial to ultimately determine the most efficacious and acceptable approaches for mentoring URM faculty. Program evaluation<br>has several important roles, including holding stakeholders accountable and highlighting programmatic areas that may need improvement.
White, Buhr and Pinheiro, 2009	Mentoring: A Key Strategy to Prepare the Next Generation of Physicians to Care for an Aging America	The author aims to provide a review of the broad research literature on mentoring as an instructional strategy and practical insights into how to improve mentoring of the physicians who will provide care to a growing population of frail elderly patients.	Not explicitly stated although it would appear that Literature Reviews draw from a constructivist perspective.	1) Purpose of mentoring - Within the context of geriatrics, mentors can fulfill three specific purposes: (1) help learners choose an area of specialty within geriatrics and recognize the importance of geriatric knowledge for most specialties, (2) help fellows and new faculty navigate advancement in the academic environment, and (3) help new physicians enter a local medical community and develop a high-quality, professionally rewarding, financially viable practice that meets the needs of older adults.<br>2) The role of the mentor - The role of mentor is a balancing act between support and challenge. Ultimately, mentors	● There are four phases of mentoring: initiation, cultivation, separation, and redefinition. Among medical students, mentors may want to start their work by focusing primarily on the first two behaviours. Mentoring needs to become an even more embedded and excellent form of teaching and learning within geriatrics education, the academic community, and the practicing geriatrics profession. For this to happen, mentoring needs to be taught, which can be done at multiple levels in the educational system.<br>● Even when few experienced mentors are

				provide vision, helping mentees see where they have been and where they are going. Six behavioural functions exist in the context of the mentor role: relationship emphasis, information emphasis, facilitative focus, confrontive focus, mentor as model and simulate vision.<br>3) The role of the mentee - The most common tasks and functions of mentees in a mentoring relationship are to assume the initiative and responsibility for setting and achieving goals, ask for advice and constructive criticism, and demonstrate openness and honesty in the relationship.<br>4) Mentoring pitfalls - Common pit- falls of the mentoring relationship include the misuse of power, charges of favouritism among mentees, excessive emotional dependence by mentor or mentee, and differing ethics.<br>5) Evaluation of mentoring - Measuring success of mentorship may be more accurately accomplished through examining the importance of pro- gram leadership, mentor selection, mentor training, mentee selection, and mentor–mentee pairing.	available, faculty physicians can form groups that provide peer mentoring.

				6) Equipping the mentor - Mentors need to be developed through formal programs that address diversity, setting objectives, process, and out-comes.<br>7) Mentoring approaches – includes informal mentoring and formal mentoring and each achieves different goals.	
McKenna and Straus, 2011	Charting a Professional Course:<br>A Review of Mentorship in Medicine	The authors aim to review the current literature for mentorship in medicine, including characteristics of successful mentors and their mentees, and the emerging role of formal institution-wide mentorship programs.	Not explicitly stated although it would appear that Literature Reviews draw from a constructivist perspective.	1) Characteristics of the successful mentor and mentee - Both mentee and mentor must equally contribute to the success of their union. Mentees must take initiative in driving their relationships, being proactive around scheduling meetings and identifying in advance topics for discussion. The ideal mentor was described in terms of 3 dimensions: personal, relational, and professional. Special attention has been paid in the literature to the mentorship needs of women and minorities.<br>2) Characteristics of the mentoring relationship - The chemistry between mentee and mentor is perceived as vital to their relationship. Harmony of common interests, both personal and professional, predicts mentorship success. The relationship should be oriented with early identification of goals, milestones, and expectations. The fundamental beneficiary of	● Institutional buy-in is necessary for widespread mentor- ship in an academic setting.<br>● Programs designed to aid junior faculty members have shown benefit for networking, grant and scholarly writing, career planning, and promotions.<br>● Formal mentorship programs must provide a venue for forming partnerships between mentors and mentees. The ability to locate appropriate mentors in a new job may prove difficult, and institutional frameworks to support mentees in identifying faculty mentors should exist.<br>● Equipping mentors with the appropriate skills to lead and facilitate the development of colleagues is an important component of a mentorship program.<br>● When developing mentorship programs,

				the relationship should be the mentee, and clear expectations regarding conflict resolution and intellectual property in the context of academic discussions must be initiated at the onset of the relationship.	academic centers should explore appropriate recognition for mentorship. A frequently cited barrier to mentorship has been a lack of “academic credit” for the time and effort it demands.
Ghawji et al, 2017	Perspectives of students and mentors on a formal mentorship program in Saudi Arabia	The authors aimed to describe the strengths, weaknesses and challenges of the mentorship program among preclinical medical students at Alfaisal University, Riyadh.	None as this is a perspective.	1) Mentee perspective - Almost half the surveyed students believed that the mentorship program was helpful in their professional growth and grade improvement. When asked about possible mechanisms to improve the overall effectiveness of the program, the suggestions included better communication initiated by mentors, such as regular meetings. Most of the students identified lack of motivation as the major hurdle in the program.<br>2) Mentor perspective - Approximately 75% of the mentors were willing to spend 1-2 hours per week on the program. The majority of mentors claimed to have met their mentees, and checked on their mentee's progress once every other month. Approximately half thought that students did not accept criticism, and failed consequently to reassess their performance. Most common suggestions were that the mentors should be provided with assessment information for mentee exams and that	● It is important to develop mentoring skills among both experienced and inexperienced mentors. The authors suggest specific mentoring training to facilitate productive mentor/mentee relationships.<br><br>● Mentees and mentors should be informed of their responsibilities and scope of their relationship.<br><br>● Mentees should develop a positive attitude towards the mentoring program early in their tenure.<br><br>● Communication and motivation on the part of mentee and mentor are the most important factors for success.

				mandatory meetings should be scheduled alongside appropriate faculty training.	
Aagaard and Hauer, 2003	A Cross-sectional Descriptive Study of Mentoring Relationships Formed by Medical Students	The authors aimed to understand how these relationships form and whether certain students were more likely to develop successful mentoring relationships.	This is a cross-sectional descriptive and analytic study.	232 students completed questionnaires.<br><br>1) Fourth-year students most commonly met their mentors during the third (29%) and first (21%) years of medical school, followed by fourth year (19%), second year (17%), before medical school (8%), and other (6%). They met their mentors during inpatient clerkships (28%), through research activities (19%), or by seeking a mentor with similar interests (23%). Less commonly, students met their mentors during outpatient clerkships (9%), or through committee/organization participation (4%).<br>2) Mentors most commonly provided personal support, role modeling, and career advising. Personal-support functions included motivation (98%), moral support (91%), and personal advice (60%). Career-advising functions included assisting with specialty (98%) and residency choice (78%) decisions and providing opportunities that aided in career advancement (83%). Eighty-nine percent of mentors served as role models for career and 80%	● Performing research either before or during medical school was highly correlated on both bivariate and multivariate analysis with having a mentor. Several studies examining the impact of a research mentor on career choice and academic success have found that mentors are associated with decisions to pursue a research career. Students with mentors tended to be interested in both research and academic careers. Students may also pursue research with the goal of developing a mentoring relationship.

				served as role models for achieving balance between personal and professional life. Less commonly, mentors provided research opportunities (60%), collaboration on research/ projects (58%), resources such as funding, office space or administrative assistance (39%), or non-research project opportunities (33%). Students met with their mentors weekly or more often (24%), monthly (29%), or less than monthly (47%). Frequency of meetings did not correlate with overall satisfaction with advising.	
Nor, Yusof and Rahim, 2017	Characteristics of mentoring programs in the early phase of medical training at the Universiti Sains, Malaysia	Mentoring programs are important elements of the personal and professional development of medical students. Mentors must focus on the real issues that students face during the mentoring process. The authors aimed to explore the need for mentoring programs for first-year medical students at the Universiti Sains, Malaysia (USM).	A qualitative case study was conducted. Purposive sampling was employed to select the study participants. Data collection was carried out using semi-structured interviews.	Interview transcripts were analysed and the themes were as follows:<br><br>1) Soft skills – This consists of 3 categories, namely time management, study skills and communication skills. Academic overview - All participants need to be exposed to the structure of the medical courses in which they will enroll.<br><br>2) Social skills – This consisted of social adjustment to the new environment and social activites (e.g for mentees to release stress)	● Mentors should be aware of mentees' expectations for guidance in the medical education program. Likewise, mentees should be reminded to express their real needs during mentoring.<br>● An academic overview was one of the primary requirements for the mentoring relationship. Mentoring activities, particularly those implemented during the early phase of medical training, can help medical students adapt to a new learning environment. A previous study also suggested that mentoring activities can

				3) Motivation from mentors – Motivation consists of morale support where mentors need to supervise the programs organised by students and personal support which functions as a source of moral support for mentees.	improve students' academic performance.<br>● Mentors could initiate social activities to encourage the development of social skills amongst their mentees. In addition, reintroducing the peer-group mentoring program during the early phase of medical training might address this concern.<br>● Mentoring is a valuable activity that benefits students, not only in terms of academic aspects, but also in terms of personal and emotional aspects (e.g, communication and affective skills, feeling supported and motivated).
Dimitriadis et al, 2012	Characteristics of mentoring relationships formed by medical students and faculty	Little is known about the characteristics of mentoring relationships formed between faculty and medical students. The authors aimed to find out the type of students are likely to seek a mentoring relationship, the expectations of mentees from the mentoring relationships, and the content of the mentor-mentee relationship.	Quantitative analysis was performed using descriptive statistics and Wilcoxon-test to assess differences in grades between students with and without mentors.	Qualitative text analysis was performed and the results were as follows:<br><br>1) Participant characteristics – This was described in terms of percentages of: students involved in mentoring programs, gender distribution and school examination results. More female than male students had a mentor, and both females and males preferred male mentors over female mentors. Students who performed better academically tended to have a mentor.	● Mentoring is a key factor for professional success in medicine.<br>● Participation in this voluntary mentoring program varied greatly with students' progress in the curriculum. The demand for mentoring decreases with the amount of experience and acquaintances a student has made in hospitals during their clinical years of study.<br>● The authors believe that the influence of mentors in formal mentoring programs is not strong in programs which are voluntary for both mentors

				2) Role of mentors – The most commonly cited roles are that of a counsellor, agent for contacts and provider of ideas.<br>3) Topics discussed in mentoring relationships – Most mentees seek advice regarding research, career planning and experiences abroad.<br>4) Communication between mentees and mentors – Most mentees and mentors met once or twice per semester. Other communications were done via email and telephone.<br>5) Satisfaction of mentors - Mentors almost unanimously felt that they had been able to help their mentees and answer their questions.<br>6) Outcomes of the mentoring relationships - Mentees stated that their mentor had most facilitated their development in the areas of career planning and research. Mentors also had a positive influence on arrangements for clinical electives, final year electives and experiences abroad.	and mentees, where mentees are free to choose their mentors and meet them as often as they need.
Farkas et al, 2019	Mentorship of US Medical Students: a Systematic Review	Mentoring of medical students remains a core pillar of medical education, yet the changing land-scape of medicine has called for new and innovative mentoring models to guide students in	Not explicitly stated though it would appear that systematic reviews stem from a positivist approach.	Our search yielded 3743 unique citations. Thirty articles met our inclusion criteria. The themes are:<br><br>1) Program description - this includes participants, mentorship model, program	• Programs with objective measures of success five used exclusive dyad mentorship which suggests the intensity of traditional dyad mentorship may result in more tangible measures of success.

		professional development, career placement, and overall student well-being. The objective of this review is to identify and describe models of mentorship for US medical students.		objectives, and program components.<br>2) Mentoring models - The most common model of mentorship used was the traditional dyad model. Other methods include traditional group mentorship, "tiered" group mentorship, multiple models of mentorship with a "vertical" approach and a combination of dyad and group mentoring.<br>3) Program objectives - Most programs stated broad objectives including assisting medical students with career development/career planning, professional development and well-being. Many of the programs had the specific objective of mentoring either underrepresented minorities (URM) or female medical students.<br>4) Program evaluation – Majority reported objective outcomes related to clerkship grades, research productivity, and residency match. Others were subspecialty specific or focused on URM students.<br>5) Program cost - Many of the studies discussed the costs of these programs. For those that were part of an advisory dean/college programs, faculty salary support was a significant	• The lack of objective outcomes and comparison data is a common theme in the mentorship literature and makes it difficult to assess the long-term impact of these mentoring programs.<br>• While the cost of several of these programs was significant, the upfront investment may be justified if mentorship can help reduce burnout, given the significant cost of having to extend medical school training. While it is unlikely that mentorship alone will reduce burnout without more comprehensive and systemic reforms, it may help improve factors associated with burnout including isolation and feelings of powerlessness.

				cost. Use of medical student mentors reduced costs.<br>6) Barriers of programs – This includes faculty busy schedules, competing demands and responsibilities in student and resident mentors, small number of URM faculty (for programs targeting URM students), and mentor skill development.	
Meinel et al, 2011	More mentoring needed? A cross-sectional study of mentoring programs for medical students in Germany	Despite increasing recognition that mentoring is essential early in medical careers, little is known about the prevalence of mentoring programs for medical students. The authors aimed to find out the prevalence of mentoring programs for medical students in Germany as well as the characteristics, goals and effectiveness of these programs.	This is a cross-sectional study. The authors designed a questionnaire. For numeric answers, mean, median, and standard deviation were determined. For free-text items, responses were coded into categories using qualitative free text analysis.	The authors received a total of 39 responses from 36 medical schools in Germany:<br><br>1) Prevalence of formal mentoring programs for medical students in Germany – 25 active programs were identified<br>2) Goals of mentoring programs - The majority of programs intend to build and expand mentees' networks at the medical school such as among faculty and peers, while others aim to enhance students' academic performance. Counseling students in difficulties was also a frequently mentioned goal.<br>3) Strategies how programs hope to achieve their goals - These included building a professional network for the mentee, providing a continuous contact person, personal support and offering an opportunity for feedback and self-reflection.	● There is great heterogeneity of declared goals of mentoring programs, and this reflects the multifaceted notion of mentoring.<br>● Further research is needed to measure the effectiveness and efficiency of mentoring for medical students and establish factors influencing the success of mentoring pro- grams. The evaluation of medical student mentoring programs should also address the benefits for mentors and the educational institution.

				4) Intended character of mentoring relationships – described as personal, early in the career of mentees, regular and long-lasting.<br>5) Mentoring models – one-on-one setting, group mentoring, voluntary or mandatory participation.<br>6) Mentees – A wide range of mentees are included, including underrepresented minority and female students.<br>7) Mentors - The median ratio of mentees per mentor is 5.9. Most programs feature faculty physicians as mentors. Fewer programs include non-faculty physicians from affiliated teaching hospitals, physicians in private practice and faculty scientists. Other programs rely on students as mentors.<br>8) Incentives for mentors – This comes in the form of financial reimbursement, recognition and wages as per working hours, reduced teaching obligations, specific training for mentors, certificates of acknowledgement, or no incentives.<br>9) Matching – Majority of programs assign mentors to mentees, while others allow mentees to choose. Assigning may occur randomly or via matching processes.	

				10) Means of communication – Personal meetings between mentors and mentees are a universal element of all 22 mentoring programs. On average, mentors and mentees meet 7 times per year. E-mail is a commonly used form of communication between mentors and mentees.<br>11) Funding and staff – 50% of programs reported receiving funding from university.<br>12) Evaluation and outcome – Majority conducted regular evaluation. Evaluations are performed through online or paper surveys, interviews or feedback meetings. Most evaluations focused on mentee and mentor satisfaction corresponding to Kirkpatrick's level 1.	
Kalen et al, 2015	Longitudinal mentorship to support the development of medical students' future professional role: a qualitative study	Mentoring has been employed in medical education in recent years, but there is extensive variation in the published literature concerning the goals of mentoring and the role of the mentor. There is still a need for a deeper understanding of the meaning of mentoring for medical students' learning and development. The authors aimed to explore	This is a qualitative study. Participants were interviewed individually about their experiences of combined group and one-to-one mentoring<br><br>Data were analysed using a latent, interpretive approach to content analysis.	Themes from the interviews were extracted:<br><br>1) Integrating oneself with one's future role as a physician – This theme was interpreted on the basis of three categories: a vision of the future as a physician, hope to manage it and combine the professional role with oneself as a person.<br>2) Experiencing clinical reality with the mentor creates incentives to learn This theme was interpreted on	• A longitudinal mentorship program can help medical students to integrate themselves as individuals with their future role as physicians. Mentoring focusing on the non- medical skills can help medical students to integrate the professional role with their own personalities and promote their understanding of the wholeness of professional competence in the process of becoming a physician.

		how formal and longitudinal mentoring can contribute to medical students' professional development.		the basis of two categories: Clinical experiences with the mentor spur motivation and Learning about medicine in the clinic.<br>3) Towards understanding professional competences of a physician - This theme was interpreted on the basis of two categories: Orientation about the physician's areas of competence and Learning about professional behaviour.	● The students' statements concerning their experience of the mentorship indicated learning and understanding, and also motivation and the creation of meaning.<br>● Individuals' personalities can be integrated in the 'personal profile,' which is the foundation for all professional activities.
Fallatah, Park, Farsi and Tekian, 2018	Mentoring Clinical-Year Medical Students: Factors Contributing to Effective Mentoring	Academic mentoring is an effective method of enhancing undergraduate medical student academic performance, research productivity, career planning, and overall satisfaction. The authors aimed to investigate the relationship between mentor characteristics and mentee academic performance, with an emphasis on identifying students who need special support.	A cross sectional study was conducted with data collected at different time points.<br><br>SPSS IBM 20 was used for the statistical analysis. Descriptive statistics were used to examine and synthesize trends in the data. Comparisons of means and proportions were conducted using t tests and chi-squared tests.	Data collected revealed the following themes:<br><br>1) Pre-program survey on mentees– Majority of students reported attending all mentoring meetings during their foundational years. Students ranked academic guidance as the most important aspect of mentoring, followed by social and psychological support and then career planning. In terms of the students' preferences for type of mentor, there was a relatively even distribution among senior faculty, junior faculty, residents, and senior medical students or interns. Students had a variety of suggestions for mentoring meeting frequencies, with most preferring monthly meetings.<br>2) Mentor characteristics – this was categorized into mentor	● Most medical students valued academic mentoring. Students thought that academic mentoring would be effective in supporting their academic performance, psychological needs and career planning.<br>● Mentor interest and motivation was an important predictor of effective mentoring. Mentor motivation had a significant impact on meeting activi- ties, student feedback, referral for special support, and, to some extent, student academic performance in the short term. This indicates the importance of mentor training.<br>● Students should be instructed about the importance and benefits of mentoring programs before

				level, department and if mentor is motivated and interested in learning.<br><br>3) Student examination results – Students who attended mentoring meetings had higher overall examination scores than those who did not attend group meetings.	starting a structured mentoring meeting.
Quek and Ong, 2018	Improving mentoring through a blend of novice and peer, near peer and group mentoring: a commentary	Mentoring is increasingly seen as a means of providing appropriate, personalised, timely and holistic support for learners. Recent reviews suggest that a particularly effective means of providing such support lies in novice mentoring or mentoring between senior clinicians and medical students or junior doctors. However shortages of mentors, time constraints both on the part of mentees and mentors and concerns about the timeliness of mentoring support have raised concerns as to the viability of novice mentoring on a larger scale.	None as this is a commentary.	1) Novice mentoring is coupled with near-peer and peer mentoring to provide timely support.<br>2) Mentoring in the palliative mentorship initiative utilises support via phone contact, email and text messaging.	● Novice mentoring achieves the dual function of encouraging peers to share resources and help each other, while providing a guide who crucially possesses the necessary professional, academic and psychosocial experience that can translate into practical advice and help.<br>● Peer mentorship usually evolved into a connection that more resembled a friendship than a mere working relationship which has been found to sustain effective mentoring relationships.<br>● The process is also mutually beneficial with senior members of the near peer mentoring dyad "developed essential attributes of time management, communication and leadership for mentors.<br>● The PMI mentoring model creates an environment in which experiential learning

					empowers mentors to make a difference and witness that change for themselves.
Y. El Miedany, 2019	Rheumatology Teaching: The Art and Science of Medical Education<br><br>Chapter 22: Mentoring in Rheumatology	Effective mentoring, as reported by residents, research faculty and residency programme directors, plays an important role in the career of physicians, particularly during residency/specialty training, and is a key to career development of research faculty in clinical translational science. This book chapter aims to discuss different categories of medical mentoring, its targets and the challenges it may face.	None as this is a book chapter.	1) Mentoring in medicine – mentoring has been shown to contribute to an individual's career development in academic medicine, clinical and non-clinical aspects, medical research, career satisfaction and perceived institutional support. Academically, the mentoring process provides a means by which junior faculty can develop professional academic skills including career management, knowledge about academic medicine and collegial networking. Clinical mentoring is a critical approach to medical training, as it provides a bridge between didactic training and independent clinical practice.<br>2) Types of mentoring – academic mentoring (has 3 roles: teaching, service and research), clinical mentoring (fosters ongoing professional development to yield sustainable high-quality clinical care outcomes) and medical research mentoring (involves a mentor who takes interest in a trainee's research career development).<br>3) Methods of mentoring – one-to-one, team mentoring, multiple mentors, peer mentoring, distance mentoring.	• Mentoring in Rheumatology - The demand for rheumatologists worldwide is increasing; however, the gap between the number of rheumatologists in training and the number needed continues to widen.<br><br>• Value of mentoring in Rheumatology - Mentoring is a reciprocal, long-term relationship with an emotional commitment that exists between a novice (protégé) and experienced (mentor) healthcare professional; mentoring implies a knowledge or competence gradient, in which the teaching–learning process contributes to a sharing of advice or expertise, role development and formal as well as informal support to influence the career of the protégé. Mentoring provides protégés and mentors with opportunities for professional growth and career satisfaction. Lack of such satisfaction with a career in medicine may contribute to healthcare

				Each of these methods have their own unique benefits.<br>4) Challenges in medical mentoring – This includes diversity (culture, background, race etc), ethics (inappropriate ethical behaviours in mentor-mentee relationship, power dynamic, mentoring abuse etc)<br>5) Problems and solutions in medical mentoring – common problems, such as mentors providing inadequate support, dealing with conflicting demands, conflicting advice, lack of commitment, and mentee or mentor neglect are highlighted. Solutions are suggested.	professional leaving the profession.<br>● The rheumatology mentoring path can be grouped into three categories: faculty–undergraduate student mentoring relation- ship, faculty–graduate student mentoring relationship and the mentoring relation- ships among faculty members.
T. W. Farrell et al., 2013	Preparing to Care for an Aging Population: Medical Student Reflections on Their Clinical Mentors Within a New Geriatrics Curriculum	Reflective writing techniques such as journaling help provide insights into the process by which medical students are men- tored and develop into practicing physicians. The authors sought to analyze medical students' journals regarding their mentored experiences within a new geriatrics curriculum at a U.S. medical school.	The authors employed qualitative analytic techniques using an interdisciplinary team process.	Three major themes emerged:<br>1) Exposure to clinical mentors challenged medical students' preconceptions regarding older adults and geriatric medicine<br>2) Students learned new medical knowledge and techniques from observing their mentors; and<br>3) Students provided positive and negative assessments of their mentors.	● Mentorship of medical students by practicing physicians is critical for students' professional development as they reflect upon clinical situations and weigh future practice options that may or may not include older adults.<br>● Medical students' journals reveal that exposure to older adults in a mentored setting may help reverse negative preconceptions about their care.
Zuzuarregui, Wu and Hohler, 2018	Promoting Careers in Neurology: Mentorship of Medical Students	Mentoring is important in order to continue to attract talented and altruistic	None as this appears to be an opinion paper.	1) Formal mentoring within Longitudinal neurology curriculum - There are numerous models for neurology education throughout the country, and	● Successful mentoring can facilitate success in the career of a mentee. This depends on several factors, but requires effort from both the

		students to careers in neurology.		longitudinal programs are ideal. The traditional curriculum allows for formal mentoring. Students have course and clerkship directors who provide them with guidance about the subject matter. Residents and faculty provide on-the-job training and mentoring during clerkships. Career mentoring about educational and research opportunities and guidance on residencies are provided. Feedback from medical students suggests that a mentoring program during clerkships provides benefits for their careers.<br>2) Inspiring students in neurology through informal mentoring programs - Informal mentoring programs provide an opportunity for more career-based discussions, optimize work–life balance discussions, and may create a framework for research and educational opportunities. Faculty serve as role models for the residents and the medical students. Students often pursue informal mentorship for career-related reasons. Studies report that students generally perceived their informal mentors positively, and the reported student's career choice was associated with the discipline of informal mentors.	mentor and mentee. Although a mentor may be able to provide avenues for productivity in research, the qualities of a mentor are equally as important to facilitate the success of the mentee's academic career.<br>● The mentees should also take initiative to find a mentor with these qualities and be assertive in obtaining advice or opportunities that facilitate their professional goals. Through this process, the foundation is set for trainees to become a successful mentor themselves.<br>● Mentoring program evaluations note great interest and high levels of satisfaction of participants. Most programs lack goals, a short- and long-term evaluation of the individual successes of the participants, and the program's cost–benefit analysis.

				3) Team approach to mentoring - Faculty, course directors, and clerkship directors serve the main roles as formal mentors. Residents are an important component for successful inspiration of the medical students, as they work with the student daily. Medical students provide peer mentoring about seeking mentors, educational support, and residency selection guidance.<br>4) Characteristics of mentors – A mentor should demonstrate admirable personal and professional qualities that the mentee can relate to, as well as strive toward in their own life.	
Bertoncelj et al., 2014	Mentoring of young professionals in the field of rheumatology in Europe:<br>results from an EMerging EUlar NETwork (EMEUNET) survey	To explore perceptions of, participation in and satisfaction with mentoring programmes among young clinicians and researchers in rheumatology in Europe. To identify mentoring needs and expectations focusing on gender-specific differences.	A survey was administered. Descriptive and summary statistics were calculated from responses based on the total number of responses per question. Chi-square or Mann-Whitney tests were used.	The following themes were revealed:<br><br>1) Perception of existing mentoring possibilities – mentoring was available during clinical and research training, as overall career mentoring and specific project mentoring. No gender differences were reported.<br>2) Participation in and satisfaction<br>with existing mentoring programmes – Majority of respondents did not participate in a mentoring programme. Existing mentoring programmes covered mentoring needs and expectations of respondents with	• Self-identification of mentors by mentees and the right interpersonal match can foster a successful mentoring relationship. organizations can facilitate this process by providing direct mentor-mentee contacts, by establishing web-based mentor-mentee networks or by developing structured mentoring programmes.

				a rating median of 6 on a 1 to 10 Likert scale. Women tended to be less satisfied than men with how mentoring programmes covered their needs and expectations. respondents being very satisfied with mentoring were significantly more likely to participate in research mentoring programmes.<br>3) Type of mentoring needed<br>for career development – Majority of respondents selected career guidance – one to one mentoring by a senior dedicated scientist over a period of years, as most beneficial for their career development.<br>4) Choice of mentor – Most stated that they would select a mentor within their own country and current institution. Majority preferred a clinical mentor.<br>5) Expectations from mentoring - Better career planning was ranked highest among the expectations from mentoring, followed by networking, personal development and informed career decision making.	
Arnold, 2005	Mentoring the Next Generation:<br>A Critical Task for Palliative Medicine	The author discusses the importance of developing the second generation of leaders and mentoring the	None as this appears to be an opinion article.	1) Mentoring the next generation - To maximize the probability our junior clinicians succeed, they need mentoring. Most successful individuals have	• We need to equip senior clinicians with the skills and time needed to mentor their junior colleagues.

		new recruits to palliative medicine.		one to four mentors. In the academic world there is clear evidence that good mentoring is associated with successful grant applications and academic publications. In the business literature, there is data that mentoring is associated with higher annual income and job satisfaction.<br>2) Importance of mentoring in palliative medicine - Mentoring is particularly important in a new field such as palliative medicine. First, the evidence base is changing rapidly. One needs teachers to help learn the cognitive, affective and behavioral skills required to be a competent palliative care doctor. This means teaching both the "formal knowledge" and the "informal art" of palliative care. In a new field, many of the pioneers know each other and thus a sponsor who can facilitate protégé's professional advancement is critical. Palliative medicine requires changing one's conceptual model from diseases and diagnosis to patient goals, prognosis and function.	● Clinical models are different from those in large medical centers and we need to make sure the mentoring processes will fit the environment. Moreover, we need to develop distance-mentoring for those clinicians who are working in jobs where there are no senior palliative care clinicians.
Ramanan et al, 2005	Mentoring and Career Preparation in Internal Medicine Residency Training	Mentoring during the early stages of a career has been associated with high career satisfaction and may guide development of	The authors developed a questionnaire, and conducted bivariate analysis. They determined statistical	329 residents responded to the survey. The themes derived were as follows:	● The authors demonstrate the importance of mentoring to personal and professional development during residency, and the

		professional expertise. Little is known about mentoring experiences during residency training. The authors aimed to describe mentoring relationships among internal medicine residents, and to examine the relationship between mentoring and perceived career preparation.	significance with Chi-squared testing for dichotomous variables and logistic regression for multivariable analyses.	1) Description of mentoring relationships – Majority of residents felt that it was important to have a mentor during residency, were satisfied with mentoring. Half of respondents reported that their mentor was assigned to them.<br>2) Positive and negative aspects of mentoring – Majority derived benefits from mentoring in terms of personal and professional development. Benefits included helpful advice on career decisions, clinical work, and research, with a smaller percentage describing assistance finding a position after residency or guidance when facing disappointment or failure. Negative aspects of mentoring included discomfort discussing ''important issues'' with their mentor and feeling as if they were an imposition on their mentor.<br>3) Characteristics of mentors – Physical and career characteristics of mentors were described.<br>4) Identification of mentor and satisfaction with mentorship - qualities of the relationship significantly associated with overall satisfaction with mentorship: importance to personal development, keeping in touch regarding progress,	association between having a mentor and the perception of excellent career preparation.<br>● Of concern, the relative lack of mentoring that identified among interns and underrepresented minority residents is a potentially serious problem, as is the finding of female residents' report of less adequate career preparation compared with their male peers.<br>● Effective mentorship—a relationship whose value has been demonstrated among faculty physicians —is highly valued by residents and can contribute to improved satisfaction with career preparation and their experience of training.

				belief that the resident was not imposing on the mentor, and having a mentor who provided thoughtful advice on research. Qualities that were not significantly associated with satisfaction with mentorship included: providing advice on clinical work, handling disappointment or failure, finding a position after residency, or having a meaningful personal relationship with their mentor.	
Mashman, 2009	Mentors in Cardiology	Mashman (2009) reflects on the mentors that he has had and the impact they have had on him.	None as this is an editor's page.	1) The author has had strong cardiology mentors who have had a profound influence not only his career and life. These relationships have evolved from teacher to mentor, to colleague, to friend.<br><br>2) A mentor is able to add wisdom, judgment, life experience, and context to the topic of education. Mentors have deeper long-term relationships with their mentees that may extend across many facets of their lives. A mentor may also help guide a pupil through personal extra- curricular challenges. This person in his or her position of authority and respect can serve as an unwitting sounding board and can give advice by proxy.<br>3) The mentoring bond has to be supported by the teacher	• Professors of cardiology should be encouraged to be mentors to their pupils. Academic training programs should find ways to reward this behaviour. The College should continue to recognize and honour giants and mortals alike who work to educate beyond the mini- mum. We should all take the time and effort to acknowledge and thank our mentors for the efforts they have ex- pended on our behalf, so that each of us will be more likely to behave similarly.

				who has a genuine desire to do so. It can be difficult to be a mentor, and being a mentor is not genetically or evolutionarily rational. There are economic pressures, time pressures, and limited resources. Potential mentors can be torn between nurturing a trainee and investing those energies in their own careers and their own families. Mentoring requires great commitment, sacrifice, and faith in the benefit of this investment.<br>4) In the short term, a mentor can gain camaraderie, companionship, and a sense of pride in helping shape a pupil's career. The mentor can get a trainee to help with his/her work, such as research tasks or teaching lower level students. Mentors can gain tremendous emotional fulfilment in having nurtured people who are destined to have strong careers, contribute to the scientific body of information, and provide good patient care.	
Jotkowitz and Clarfield, 2006	Mentoring in internal medicine	Mentoring is a relationship aimed at fostering the development of the less experienced person. The internal medicine community has recently recognized the importance of this relationship in the advancement of careers in the profession. The authors	None as this appears to be a commentary on the benefits of mentoring.	1) Faculty members with mentors were better prepared for a research career and reported greater career satisfaction than those without. Mentors were able to assist the mentee with publications, grants, networking, and "politicking". Successful mentoring relationships appear to	● Mentoring seems to have a special significance in relationship to women faculty and those from minorities. Studies have shown that a lack of mentoring is one of the main reasons for the low numbers of women in senior faculty positions.

		encourage academic institutions to help forge these relationships by developing their own formal systems of mentoring and to evaluate and report their efforts.		be based on empathy, trust, and honesty.<br>2) Participants in a collaborative mentoring program were better able to identify their core values, improve their skills in career planning, develop collaborative relationships, improve their skills in scholarly writing and oral presentation, and have a greater satisfaction with academic medicine.	● The authors encourage academic institutions to help forge these relation- ships by developing their own formal systems of mentoring and to evaluate and report their efforts.<br>● A crucial aspect of mentoring success is the mentee's initiative in finding a suitable advisor. Mentoring can encouraged by having it serve as an explicit criterion for promotion. The effort could be documented via joint publications with junior faculty and/or having to receive letters of recommendation from a mentee, in a way reversing the usual direction of such relationships.<br>● Faculties can designate “Mentor of the Year” awards that can also help in promotion, as is the case for teaching in many faculties.<br>● Institutions can sponsor faculty development sessions on how to mentor.

Appendix 3: PRISMA Flowchart

Database search:

(Mentor*) AND (Medicine* OR Medical*) AND Review* AND English AND (Physician* OR Resident* OR Facult* OR Student* OR Relation*) from Year 2000 to 2019

- PubMed 6386
- Embase 6962
- PsycInfo 1259
- ERIC 385
- Cochrane 176
- Google Scholar 213
- Scopus 2954
- GreyLit 3
- OpenGrey 31
- [illegible]

Removed duplicates

18915 title abstracts

Excluded non-relevant articles based on title and abstract (i.e. 'Mentor' not a major topic, appears only once or twice in the article)

62 full-text articles

Excluded articles based on exclusion criteria

- Allied health specialties such as Pharmacy, Dietetics, Chiropractic, Midwifery, Podiatry, Speech Therapy, Occupational and Physiotherapy
- Medical and Surgical specialties such as Surgery, Paediatrics, Emergency Medicine, Obstetrics and Gynaecology, Clinical and Translational Science, Veterinary, Dentistry
- Peer, near-peer, patient and youth mentoring
- Mentoring for leadership purposes, such as for head of departments
- Studies about supervision, coaching, role modelling, advisor, and sponsorship

41 reviews included

Appendix 4. Desirable Traits of Mentees

**Personal Traits**	Refs	**Professional Traits**	Refs
Virtues<br>● Flexible<br>● Humble<br>● Appreciative<br>● Honest<br>● Reliable<br>● Respectful<br><br>Interest in Learning<br>● Teachable spirit<br>● Self-aware<br>● Committed<br>● Motivated to learn<br>● Responsible<br>● Open-minded<br>● Reflective	(50, 52, 53)	Self-Improvement<br>● Willing to discuss flaws<br>● Ask for advice<br>● Accept criticism<br>● Learn from their mistakes<br>● Open to change<br>● Passionate about succeeding<br><br>Work Ethic<br>● Shows initiative<br>● Exhibits ownership<br>● Proactive in cultivating relationships with mentors<br>● Settling meeting agendas<br>● Meeting deadlines<br>● Made aware of codes of conduct and the mentoring framework<br><br>Professionalism<br>● Professional<br>● Ethical	(18, 50, 53)

Appendix 5. Desirable Traits of Mentors

**Personal Traits**	Refs	**Professional Traits**	Refs
Virtues<br>● Trustworthy<br>● Honest<br>● Motivated<br>● Committed<br>● Compassionate<br>● Self-aware<br>● Mature<br>● Altruistic<br>● Reliable<br>● Sincere<br>● Trustworthy<br>● Understanding<br>● Responsive<br>● Patient<br>● Respectful<br>● Motivator<br>● Positive Attitude<br><br>Connecting with Mentees<br>● Empathetic<br>● Approachable<br>● Able to connect<br>● Collegial<br>● Friendly<br>● Open<br>● Culturally sensitive	(50-53)	General Professional Characteristics<br>● Knowledgeable<br>● Accomplished<br>● Respected<br>● Influential<br>● Authorities in their field<br>● Thought Leaders<br>● Significant Academic Experience<br>● Good communication skills<br>● Good research skills<br><br>Specific to Being a Mentor<br>● Secure in his position<br>● Responsive to evolving needs of their mentees<br>● Believes in his/her mentee's abilities<br>● Brings out the best in his/her mentee<br>● Genuinely interested and passionate about developing his/her mentee<br>● Allows his/her mentee to reach full potential	(18, 50-53)

● Personal<br>● Available<br>● Non-judgemental listening		● Allows his/her mentee to be challenged<br>● Able to rejoice in his/her mentee's success<br>● Does not feel threatened or vying with mentee for credit<br>● Does not push for own agenda<br>● Actively seeking out mentees to evaluate their progress<br>● Supports mentee morally and emotionally<br>● Have best interests of mentee at heart<br>● Derives joy from mentoring<br>● Not authoritative<br>● Acknowledges mentees' contributions<br>● Nurtures mentees<br>● 'Proactive' in addressing any potential stressors<br>● 'Protects' them from adverse influences or harsh interactions<br>● Provide regular, constructive, appropriate, timely and objective feedback<br>● Cultivates emotional safety<br>● Proven mentoring track records<br>● More able to provide guidance and professional and research support to mentees<br>● Advocates for mentees<br>● Provides mentees with opportunities to network<br>● Made aware of codes of conduct and the mentoring framework<br>● Is generous in their commitment of time and energy<br>● Provides career advice<br>● Role model<br>● Acts as sponsors	
